# Directed Regeneration of Osteochondral Tissue by Hierarchical Assembly of Spatially Organized Composite Spheroids

**DOI:** 10.1002/advs.202103525

**Published:** 2021-11-21

**Authors:** Jinkyu Lee, Seoyun Lee, Seung Jae Huh, Byung‐Jae Kang, Heungsoo Shin

**Affiliations:** ^1^ Department of Bioengineering Institute for Bioengineering and Biopharmaceutical Research Hanyang University 222 Wangsimni‐ro, Seongdong‐gu Seoul 04763 Republic of Korea; ^2^ BK21 FOUR Human‐Tech Convergence Program Hanyang University 222 Wangsimni‐ro, Seongdong‐gu Seoul 04763 Republic of Korea; ^3^ Department of Veterinary Clinical Sciences College of Veterinary Medicine and Research Institute for Veterinary Science BK21 FOUR Future Veterinary Medicine Leading Education and Research Center Seoul National University 1 Gwanak‐ro, Gwanak‐gu Seoul 08826 Republic of Korea; ^4^ BK21 FOUR Education and Research Group for Biopharmaceutical Innovation Leader Hanyang University 222 Wangsimni‐ro, Seongdong‐gu Seoul 04763 Republic of Korea; ^5^ Institute of Nano Science and Technology Hanyang University 222 Wangsimni‐ro, Seongdong‐gu Seoul 04763 Republic of Korea

**Keywords:** 3D printing, composite spheroid, microchamber, osteochondral tissue regeneration, tissue engineering

## Abstract

The use of engineered scaffolds or stem cells is investigated widely in the repair of injured musculoskeletal tissue. However, the combined regeneration of hierarchical osteochondral tissue remains a challenge due to delamination between cartilage and subchondral bone or difficulty in spatial control over differentiation of transplanted stem cells. Here, two types of composite spheroids are prepared using adipose‐derived stem cells (hADSCs) and nanofibers coated with either transforming growth factor‐*β*3 or bone morphogenetic growth factor‐2 for chondrogenesis or osteogenesis, respectively. Each type of spheroid is then cultured within a 3D‐printed microchamber in a spatially arranged manner to recapitulate the bilayer structure of osteochondral tissue. The presence of inductive factors regionally modulates in vitro chondrogenic or osteogenic differentiation of hADSCs within the biphasic construct without dedifferentiation. Furthermore, hADSCs from each spheroid proliferate and sprout and successfully connect the two layers mimicking the osteochondral interface without apertures. In vivo transplantation of the biphasic construct onto a femoral trochlear groove defect in rabbit knee joint results in 21.2 ± 2.8% subchondral bone volume/total volume and a cartilage score of 25.0 ± 3.7. The present approach can be an effective therapeutic platform to engineer complex tissue.

## Graphical abstract

The spontaneously differentiated chondrogenic or osteogenic composite spheroids incorporating growth factors‐engineered fibers are spatially positioned onto each cell‐adhesive 3D printed microchamber, and the chambers are then hierarchically assembled for the biphasic construct to mimic an osteochondral tissue structure. The biphasic construct is then transplanted onto an osteochondral defect for in vivo analysis.

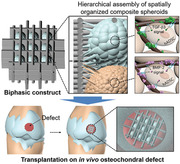

## 1 Introduction

Tissue engineering has achieved significant progress during the past few decades as a paradigm capable of reconstructing damaged organs, in particular, for potentially curing patients with osteochondral disorders.^[^ ^1^, ^2^ ^]^ The osteochondral tissue is composed of cartilage and subchondral bone, which are linked biologically and functionally to each other, and this functional complexity and hierarchical structure often hinder recovery after damage.^[^ ^3^ ^]^ Common treatment includes the use of defect‐specific 3D‐printed biomaterials engineered with instructive biomolecules and cells that can adjust the size and porosity of scaffolds according to shape of defects.^[^ ^4^ ^]^ Previous studies using 3D‐printed scaffolds have reported regeneration of cartilage or bone defect; for example, a 3D‐printed polycaprolactone (PCL) scaffold with mesenchymal stem cells (MSCs) and osteoinductive deferoxamine was studied for regeneration of a critical‐sized bone defect,^[^ ^5^ ^]^ and a silk/fibrin and gelatin scaffold with bone marrow‐derived stem cells (BMSCs) cultured in a chondrogenic in vitro environment was used for cartilage reconstruction.^[^ ^6^ ^]^ Although these previous approaches enhanced the regeneration of each target tissue, it remains challenging to achieve simultaneous distinct structural and functional characteristics of cartilage and subchondral bone.^[^ ^7^, ^8^ ^]^

To mimic the hierarchical structure of osteochondral tissue, a biphasic scaffold consisting of two layers has been studied. In general, soft materials engineered with chondroinductive molecules have been used for cartilage regeneration,^[^ ^9^ ^]^ while subchondral bone formation is induced with the use of hard synthetic polymers or ceramics engineered with osteoinductive molecules.^[^ ^10^, ^11^ ^]^ However, delamination of the two layers often causes tissue disjunction between the regenerated cartilage and subchondral bone, and the rapid infiltration of connective tissues (CT) within the construct hinders the overall regeneration capacity.^[^ ^10^, ^11^, ^12^, ^13^ ^]^ Alternatively, the loading of cells on biphasic scaffolds increases the secretion of inductive cytokines and allows for stronger interface formation through cell–cell interactions, resulting in improved and effective osteochondral tissue repair.^[^ ^14^ ^]^ For example, chondrocytes (or stem cells) and pre‐differentiated osteogenic cells can be seeded onto the cartilage and subchondral bone layer, respectively, and cultured with tissue‐engineered 3D osteochondral tissue until in vivo transplantation.^[^ ^15^, ^16^ ^]^ Moreover, chondrogenic and osteogenic cells can be bio‐printed within a one‐step synthesized 3D scaffold to prevent delamination of layers.^[^ ^17^ ^]^ The aforementioned approaches enhance the regeneration efficacy relative to the use of biphasic scaffolds without cells. However, the reconstruction of host‐like osteochondral tissue is difficult due to several problems including the loss of cells during cell seeding, compromised cell viability within a bio‐printed structure, immune rejection of pre‐differentiated cells, difficulty adjusting medium composition for co‐culturing chondrogenic and osteogenic cells, and/or undesired dedifferentiation.^[^ ^16^, ^17^, ^18^, ^19^ ^]^

Multicellular aggregates (or spheroids) from various cell types have been studied as a tissue engineering module of several target organs including cardiovascular, nerve, skin, and musculoskeletal tissues.^[^ ^20^ ^]^ Spheroids are formed by spontaneous cellular assembly, mimicking a natural tissue‐like microenvironment with elevated paracrine signaling and cell–extracellular matrix (ECM) interactions.^[^ ^21^ ^]^ A large 3D tissue can be formed by self‐assembly of spheroids using omnidirectional cell‐to‐cell interactions between adherent surficial cells, and the inner cells spread homogeneously within the engineered tissue through spontaneous ECM remodeling and cell migration.^[^ ^22^ ^]^ Moreover, the spheroids have been hierarchically arranged to recapitulate the shape of a specific host tissue structure. For example, the spheroids can be hooked by needles (Kenzan method), encapsulated within a hydrogel, and bio‐printed within polymeric scaffolds.^[^ ^23^ ^]^ However, the construct often revealed diffusion limitation because the surficial cells on the 3D tissue are compacted densely, causing issues such as difficulty in controlling the differentiation of inner cells and limited regeneration after in vivo implantation.^[^ ^24^ ^]^ The diffusion limitation within spheroids can be mitigated by a dynamic culture system using bioreactors^[^ ^25^ ^]^ or hybridized spheroid formation with microsized particles or nanofibers.^[^ ^26^ ^]^ Recently, we reported composite stem cell spheroids fabricated with ECM‐mimicking fragmented fibers (100 µm of length) immobilized with cell‐instructive biomolecules. The fibers incorporated within spheroids partially mitigated diffusion limitation and induced differentiation of stem cells into a specific lineage under general media by effective and homogeneous delivery of instructive signals to stem cells within the spheroid.^[^ ^27^, ^28^ ^]^

Taken together, the current investigations using spheroids and 3D scaffolds improve the regeneration of individual cartilage or bone tissue. However, the absence of techniques to direct chondrogenic and osteogenic differentiation of stem cells within a 3D structure in a spatially controlled manner seems to have impeded the reconstruction of osteochondral tissue. In this study, we hypothesized that the inductive growth factors provided by ECM‐mimicking fibers within the spheroid could induce the chondrogenic or osteogenic differentiation of stem cells, and spatially arranged spheroids could be fused to form a strong interface mimicking osteochondral tissue without delamination. The viability and spontaneous chondrogenic or osteogenic differentiation of the cells within the engineered spheroids cultured in a general growth medium were investigated by in vitro analysis. In addition, the controlled differentiation of stem cells within spheroids and delamination on the interface of the biphasic construct were assessed. Finally, the spatially and functionally directed 3D tissue‐engineered construct was transplanted in vivo onto an osteochondral defect and revealed significantly enhanced osteochondral tissue regeneration in native cartilage and bone.

## 2 Results

**Scheme 1:**
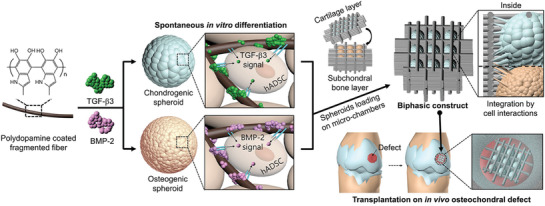
Schematic illustration of the formation of chondrogenic or osteogenic composite spheroids and the fabrication of biphasic construct positions with the spheroids for transplantation. Growth factors were immobilized on the fragmented fibers, which were hybridized with stem cells to form each type of spheroid. The spheroids were positioned hierarchically on each well of a 3D‐printed microchamber to form biphasic layers. The biphasic construct was transplanted onto an osteochondral defect for in vivo analysis.

In this study, we developed a platform combining approaches that position osteogenic and chondrogenic composite spheroids (bottom‐up) onto a 3D‐printed microchamber (top‐down) to prepare a biphasic construct (**Scheme** **1**). The construct, which structurally and functionally mimics natural osteochondral tissue, showed successful in vitro differentiation of stem cells for each osteogenic or chondrogenic lineage without dedifferentiation and enhanced in vivo tissue regeneration after transplantation. All the sample codes of composite spheroids used in this study are summarized in **Table** **1**.

**Table 1:** Sample codes of the composite spheroids

Sample code	hADSCs spheroids	w/BMP‐2 immobilized fibers	w/TGF‐*β*3 immobilized fibers	BMP‐2 diluted medium	TGF‐*β*3 diluted medium
PS	O	X	X	X	X
PS/B	O	X	X	O	X
PS/T	O	X	X	X	O
BS	O	O	X	X	X
TS	O	X	O	X	X

### 2.1 Preparation of Composite Spheroids

**Figure 1:**
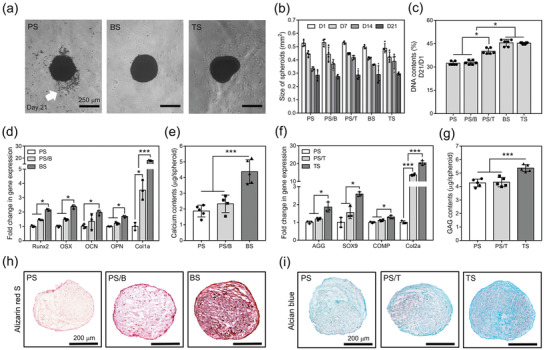
Preparation of osteogenic or chondrogenic composite spheroids. a) Phase‐contrast images of PS, BS, and TS cultured for 21 d in vitro (white arrow: cell and fiber debris, scale bar = 250 µm). b) Size change of the spheroids over 21 d (*n* = 6). c) Relative DNA contents of the spheroids after 21 d compared with those of day 1 (*n* = 6). d) Osteogenic gene expression of hADSCs from PS, PS/B, and BS (*n* = 3) and e) the amount of deposited calcium ions from each spheroid after 21 d (*n* = 5). f) Chondrogenic gene expression of hADSCs from PS, PS/T, and TS (*n* = 3) and g) the amount of GAGs from each spheroid after 21 d (*n* = 5). h) Alizarin red S staining of the cross‐sectioned spheroids of PS, PS/B, and BS and i) alcian blue staining of the cross‐sectioned spheroids of PS, PS/T, and TS (scale bar = 200 µm). All statistical analyses were performed by one‐way analysis of variance (ANOVA). * = *p* < 0.05 and *** = *p* < 0.001.

The field‐emission scanning electron microscopy (FESEM) images of the polydopamine (PD)‐coated fragmented fibers (PF) and the bone morphogenetic protein 2 (BMP‐2) or transforming growth factor‐beta 3 (TGF‐*β*3)‐immobilized fibers (BF and TF, respectively) demonstrated that the morphologies of fibers were not changed after coating (Figure S1a, Supporting Information), and 94.9 ± 2.7% of BMP‐2 and 95.7 ± 0.2% of TGF‐*β*3 fibers were coated successfully (Figure S1b, Supporting Information). The X‐ray photoelectron spectra (XPS) of the fibers revealed carbon (C1s, 288 eV), oxygen (O1s, 533 eV), and nitrogen (N1s, 399 eV) peaks for the fibers, while sulfur (S2p, 163 eV) was found only on BF and TF (Figure S1c, Supporting Information). The high‐resolution of the N1s peak for the fibers demonstrated that N1s was detected more clearly in the BF and TF than in the PF (Figure S1d, Supporting Information), and that the S2p peak was found on each BF and TF, but not on PF (Figure S1e, Supporting Information). Each type of fiber (PF, BF, and TF) was mixed with human adipose‐derived stem cells (hADSCs) to obtain composite spheroids, which were termed PS, BS, and TS, respectively. Furthermore, the PS was cultured in BMP‐2 or TGF‐*β*3 diluted media (PS/B and PS/T, respectively) as a control group. The phase‐contrast images showed that the spheroids retained their spherical shape until 21 d, but debris of cells and fibers was found on the PS but not the BS and TS (**Figure** **1**a). The sizes of PS, PS/B, PS/T, BS, and TS decreased at 21 d and were not significantly different among the groups at any time point (Figure 1b). A DNA assay demonstrated that 45.5 ± 2.2% and 45.3 ± 0.6% of DNA from BS and TS was retained after 21 d, respectively, but only 32.4 ± 1.2%, 32.9 ± 1.3%, and 40.1 ± 1.9% of DNA was retained for PS, PS/B, and PS/T, respectively (Figure 1c). Hematoxylin and eosin (H&E) staining showed that the cells and fibers in all composite spheroids were homogeneously distributed at day 3 (Figure S2a, Supporting Information), but the PS, PS/B, and PS/T showed disconnected membranes featured as unstained areas (black arrows) in each spheroid; these were not found in BS or TS after 21 d (Figure S2b, Supporting Information). Furthermore, the SEM images confirmed the densification of all spheroids over 21 d (Figure S2a,b, Supporting Information). However, the PS revealed an undensified surface structure where incorporated fibers were exposed and visualized on the surface of the spheroid (Figure S2b, Supporting Information).

The osteogenic gene expression of stem cells from PS, PS/B, and BS demonstrated that greater gene expression in PS/B than PS except for collagen type 1a (Col1a) (Figure 1d). On the other hand, all gene expression of the cells from BS were significantly more enhanced than those of both PS and PS/B; for example, the osteocalcin (OCN) expression of cells from PS/B and BS was 1.3 ± 0.1 and 2.0 ± 0.2 fold higher, respectively, and the Col1a expression was 4.2 ± 0.1 and 6.6 ± 0.2 fold higher than those of PS, respectively (Figure 1d). The deposited calcium content with a similar tendency was 4.4 ± 0.8, 2.5 ± 0.7, and 2.1 ± 0.7 µg in BS, PS/B, and PS, respectively (Figure 1e). The investigation on chondrogenic gene expression of hADSCs from PS, PS/T, and TS demonstrated that cells from TS had a significantly more enhanced expression for all genes than did the other groups (Figure 1f). For example, the aggrecan (AGG) and collagen type 2a (Col2a) expression of the cells from TS was 2.1 ± 0.3 and 20.4 ± 1.3 times greater than those of PS, respectively, and those of PS/T were 1.2 ± 0.1 and 13.7 ± 0.5 times greater. A dimethylmethylene blue (DMMB) assay showed amounts of glycosaminoglycans (GAGs) of 5.4 ± 0.3, 4.3 ± 0.4, and 4.3 ± 0.3 µg in TS, PS/T, and PS, respectively (Figure 1g). Alizarin red S and alcian blue staining demonstrated a strong intensity of reddish and blue color throughout the whole sectioned area of the spheroid in BS and TS, while the stained colors of PS/B and PS/T were greater than those of PS, but mainly were deposited on the surface of each spheroid (Figure 1h,i). Taken together, these findings indicate that, compared with PS/B and PS/T, BS and TS showed the greater osteogenic and chondrogenic differentiation despite being cultured in a general growth medium that did not contain differentiation supplements.

### 2.2 Preparation of 3D‐Printed Microchambers and Positioning of Spheroids

**Figure 2:**
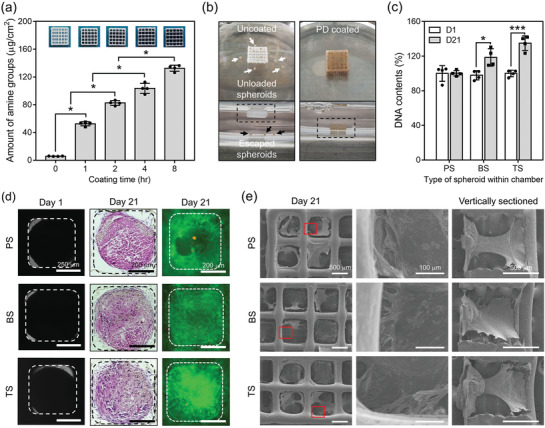
Preparation of 3D‐printed microchambers and positioning of spheroids. a) Optical images of chambers and the number of amine groups from the microchambers depending on PD coating time (*n* = 4). b) Optical images of each microchamber with or without PD coating after dipping the chambers into media and loading the spheroids (scale bar = 4 mm). c) DNA content of the cells in each PS, BS, and TS loaded within chamber at days 1 and 21 (*n* = 4). d) Phase‐contrast images of a well organized with each PS, BS, and TS at day 1 (scale bar = 250 µm), and H&E staining and live and dead staining of each group after 21 d (scale bar = 200 µm). e) SEM images of the groups from the top view (scale bar = 500 µm), the portions with red boxes were enlarged in high magnification (scale bar = 100 µm), and their vertically cross‐sectioned images (scale bar = 500 µm). All statistical analyses were performed by one‐way analysis of variance (ANOVA). * = *p* < 0.05 and *** = *p* < 0.001.

The amount of PD coated on the surface of the microchamber was increased and the color of the chamber became darker with coating time (**Figure** **2**a). The FESEM images of the chambers showed that the morphologies of lattice wells and strands were not changed after PD coating, but homogeneously distributed PD particles were detected on the surface of the PD‐coated chamber in the high‐magnified image (Figure S3a, Supporting Information). The XPS spectrum demonstrated that the carbon (C1s, 288 eV) and oxygen (O1s, 533 eV) peaks were common, while the nitrogen (N1s, 399 eV) peak was generated only after PD coating, and the high‐resolution carbon spectrum showed the C—N bond (286.0 eV) only on the surfaces of the PD‐coated chambers (Figure S3b, Supporting Information). The hydrophobic PCL chamber became hydrophilic with PD coating because the chamber was immersed in media. Thus, stable positioning of spheroids onto each chamber was achieved easily without spheroid escape (Figure 2b). In contrast, the spheroids were hard to load within the uncoated hydrophobic PCL chamber floating on the medium, and the unloaded spheroids collected at the bottom of the culture plate (Figure 2b). The spheroids of PS, BS, and TS were loaded within PD‐coated microchamber and cultured for 21 d. The DNA content increased by 119.1 ± 8.3% and 133.2 ± 6.6% in the group with BS and TS after 21 d, respectively, while that of PS increased by 102.6 ± 9.2% (Figure 2c). The phase‐contrast images of PS, BS, and TS cultured within chambers indicated that each spheroid initially fit each well of the chamber, and H&E staining revealed that the cells from each spheroid were spread toward the borderlines of a chamber and filled the spaces after 21 d (Figure 2d). Furthermore, the majority of cells in a chamber showed positive viability regardless of group (Figure 2d). The SEM images demonstrate filopodia of sprouting cells from a spheroid bound to the wall of the chamber, and each well was occupied with proliferating cells, which also migrated along the strands of the chamber (vertically sectioned images) (Figure 2e). The images of vertically sectioned samples revealed that the cells from a spheroid, which initially was positioned on the supporting strands in a well of the chamber and then migrated and proliferated along the wall to the top of the chamber over 21 d (Figure 2e).

### 2.3 In Vitro Differentiation of the hADSCs from Spheroids Positioned within the Chambers

**Figure 3:**
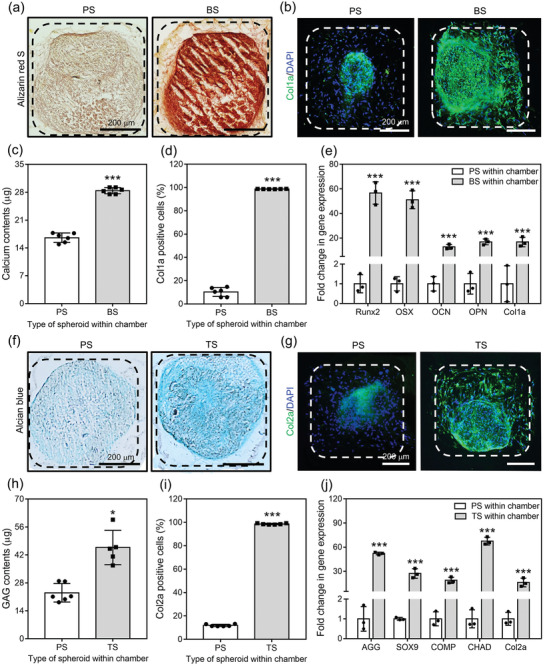
In vitro differentiation of hADSCs from spheroids within the chambers. For osteogenic differentiation, a) alizarin red S and b) Col1a IHC staining of horizontally cross‐sectioned chambers carrying PS or BS (dotted lines: borderline of chamber well, scale bar = 200 µm). c) Deposited calcium contents and d) Col1a‐positive cells from each PS and BS loaded chamber (*n* = 6). e) Osteogenic gene expression of cells from the groups (*n* = 3). For chondrogenic differentiation, f) alcian blue and b) Col2a IHC staining of horizontally cross‐sectioned chambers carrying PS or TS (dotted lines: borderline of chamber well, scale bar = 200 µm). c) Amounts of GAGs and d) Col2a‐positive cells from each PS and TS positioned chamber (*n* = 6). e) Chondrogenic gene expression of the cells from the groups (*n* = 3). The statistical analyses for PCR investigations were performed by one‐way analysis of variance (ANOVA), and the others were by Student's *t*‐test. * = *p* < 0.05 and *** = *p* < 0.001.

Alizarin red S and Col1a staining of cross‐sectioned samples culturing PS and BS within chambers demonstrated more intense calcium mineral deposition and Col1a‐positive cell staining in BS than PS (**Figure** **3**a,b). Quantitatively, 28.4 ± 0.7 µg of calcium minerals were deposited and 98.7 ± 0.1% of cells were positive for Col1a in the group cultued with BS, while those of PS were 16.6 ± 1.2 µg and 10.6 ± 4.6%, respectively (Figure 3c,d). Consistent with staining results, the osteogenic gene expression of hADSCs was more significantly enhanced in BS group than PS group; for example, the osterix (OSX) and Col1a expression of the cells from BS was 51.1 ± 7.3 and 16.7 ± 3.9 fold greater than those of PS, respectively (Figure 3e). In chondrogenic differentiation, alcian blue and Col2a staining revealed stronger staining for GAGs and Col2a cells in the group cultured with TS than that with PS (Figure 3f,g). Quantitative analysis confirmed that 45.7 ± 8.6 µg of GAGs and 98.6 ± 0.6% of cells were positive for Col2a in TS, while those in PS were 28.2 ± 3.0 µg and 11.9 ± 1.0%, respectively (Figure 3h,i). Similarly, chondrogenic gene expression of hADSCs was significantly enhanced in TS group compared with PS group, with expression of AGG and Col2a in the cells from TS at 52.1 ± 1.6 and 16.1 ± 5.3 fold greater than in those of PS, respectively (Figure 3e). Similar to the gene expression results, the protein secretion showed that the significantly greater amount of BMP‐2 (44.7 ± 4.7 ng) and TGF‐*β*3 (25.5 ± 0.0 ng) was observed from spheroids incorporating the fibers with BMP‐2 (Figure S4a, Supporting Information) and TGF‐*β*3 (Figure S4b, Supporting Information) during 21 d relative to PS group (12.5 ± 1.0 and 0.4 ± 0.0 ng), respectively.

### 2.4 Preparation of Biphasic Construct

**Figure 4:**
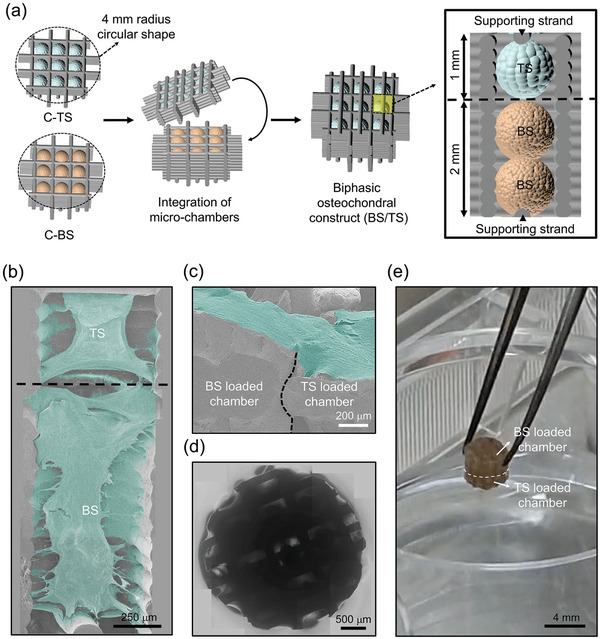
In vitro integration of BS and TS organized microchambers to form a biphasic structure. a) Schematic illustration of the process to integrate the chambers carrying BS or TS, and the illustration of spheroids positioned inside an integrated well. SEM images of b) vertically cross‐sectioned (scale bar = 250 µm) and c) outside of BS/TS (scale bar = 500 µm) (green: cells; black dotted line: borderline between chambers). d) Phase‐contrast image of BS/TS from the top view (scale bar = 500 µm). e) Optical image of BS/TS being held out of culturing medium by forceps (white dotted line: borderline between chambers, scale bar = 4 mm).

A schematic illustration explains how the chambers cultured with BS and TS were hierarchically integrated to prepare a biphasic construct (BS/TS) (**Figure** **4**a). We placed two BS or one TS within each well of the chamber with a height of 2 or 1 mm for potential bone or cartilage regeneration, respectively, and the wells were placed facing each other (Figure 4a). The FESEM image of vertically cross‐sectioned BS/TS showed that the cells from BS and TS (colored green) migrated along the walls of each chamber and made contacts at the borderline of each chamber (black dotted line). In addition, the two initially loaded BS were fused as a large cellgregate with interconnected regions (Figure 4b). Furthermore, the outside view of the FESEM image of the BS/TS showed that the proliferating cells tended to cover the outer wall of the 3D construct without disconnection between layers after prolonged in vitro cultivation (Figure 4c). The phase‐contrast image from the top view of the 3D construct showed that the BS and TS were stacked together in rows, while the wells were confluent with spreading and migrating cells from the spheroids (Figure 4d). Finally, the 3D construct was removed from the medium while picking up the only chamber carrying BS, but the construct was not separated because the cells sprouted and bridged within the two chambers (Figure 4e).

### 2.5 In Vitro Differentiation of hADSCs within the Construct

**Figure 5:**
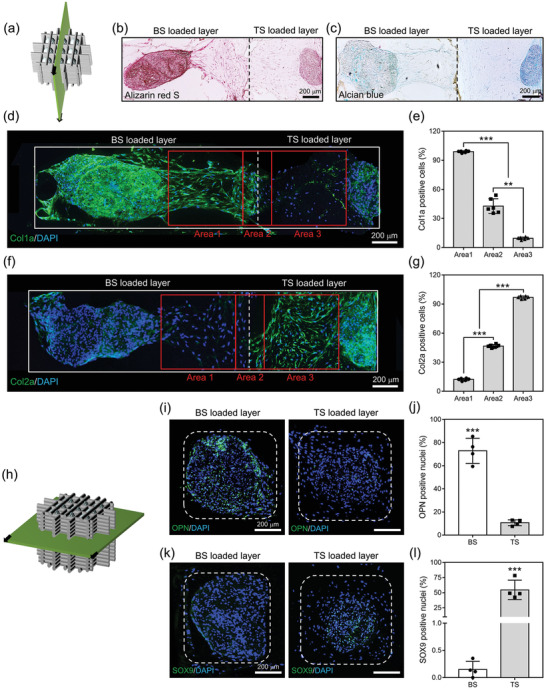
In vitro differentiation of stem cells within biphasic construct. a) Schematic illustration of the process in a vertical cross section of BS/TS. b) Alizarin red S and c) alcian blue staining of vertically cross‐sectioned BS/TS (dotted line: borderline between BS and TS loaded microchambers, scale bar = 200 µm). d) Col1a IHC staining of vertically sectioned BS/TS (scale bar = 200 µm) and e) the ratio of Col1a‐positive cells in the red boxes (*n* = 6), and f) Col2a IHC staining of vertically cross‐sectioned BS/TS (scale bar = 200 µm) and g) the ratio of Col2a‐positive cells in the red boxes (*n* = 6) (Area 1: cells spread from BS, Area 2: cells near the borderline, Area 3: cells spread from TS). h) Schematic illustration of a horizontal cross section of BS/TS. i) OPN IHC staining of horizontally cross‐sectioned BS/TS and j) the ratio of OPN‐positive cells (*n* = 4), and k) SOX9 IHC staining of horizontally sectioned BS/TS and i) the ratio of SOX9‐positive cells (*n* = 4) (dotted line: a chamber well). The statistical analyses for the ratio of Col1a and Col2a positive cells were performed by one‐way analysis of variance (ANOVA), and the others were by Student's *t*‐test. * = *p* < 0.05, ** = *p* < 0.01, and *** = *p* < 0.001.

As shown in **Figure** **5**a, the intensity of alizarin red S staining of vertically cross‐sectioned BS/TS faded from left to right (the direction of positioning two BS to that of TS). The cells within the chamber carrying BS were stained red, the sprouting cells appeared reddish but with reduced intensity, and those of TS were stained weakly (Figure 5b). Alcian blue stain showed the opposite tendency in that the TS showed strong blue staining, the spreading cells were brighter blue color, and those of BS were stained weakly (Figure 5c). The collagen‐immunohistochemistry (IHC) staining of BS/TS clearly distinguished the distinct characteristics of BS and TS such that the majority of the cells in BS was positive for Col1a (Figure 5d), and 99.5 ± 0.4% of spreading cells in Area 1 were positive for Col1a, while only 9.5 ± 1.9% of cells in Area 3 from TS were stained (Figure 5e). Meanwhile, the majority of cells in TS was positive for Col2a (Figure 5f), with 97.2 ± 1.7% of migrating cells in Area 3 stained though few cells in Area 1 were stained (Figure 5g). The cells in the intermediate area (Area 2) revealed a middle propensity, 43.1 ± 9.8% were stained with Col1a and 46.7 ± 2.4% were stained with Col2a (Figure 5e,g). To confirm the osteogenic or chondrogenic lineage of the cells in the chambers containing BS or TS, the 3D construct was cross‐sectioned horizontally for IHC staining (Figure 5h). As a result, cells in the BS area were stained for osteogenic genes (72.9 ± 10.9% positive for osteopontin, OPN) (Figure 5i,j), while those in TS revealed chondrogenicity (54.7 ± 16.1% positive for SRY‐box transcription factor 9, SOX9) (Figure 5k,l).

### 2.6 In Vivo Implantation of Spheroid‐Laden 3D Biphasic Constructs onto Rabbit Osteochondral Defects

**Figure 6:**
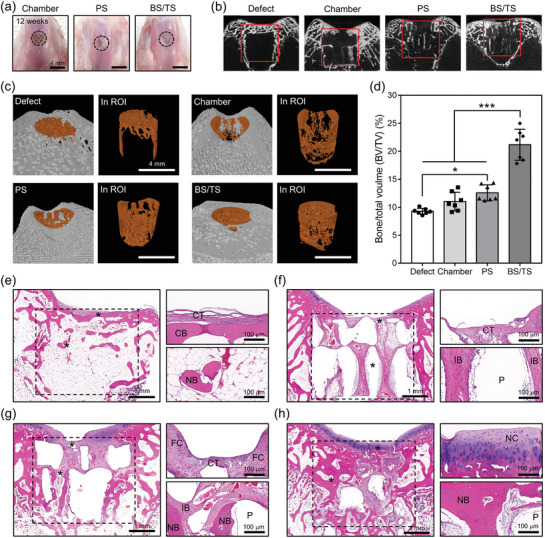
In vivo analysis after transplantation of spheroid‐laden biphasic construct. a) Optical images of harvested osteochondral tissues from Chamber, PS, and BS/TS groups (scale bar = 4 mm). b) X‐ray images (red boxes indicate defect area) and c) 3D images from *μ*CT analysis of the Defect, Chamber, PS, and BS/TS groups (region of interest, ROI, indicating defect area is colored in orange, scale bar = 4 mm). d) BV/TV values from *μ*CT analysis of the groups (*n* = 7), and the statistical analysis was performed by one‐way analysis of variance (ANOVA) (* = *p* < 0.05 and *** = *p* < 0.001). H&E staining of e) Defect, f) Chamber, g) PS, and h) BS/TS groups (scale bar = 1 mm) (the portions with “*” are in high magnification (scale bar = 100 µm); dotted line, defect area; CT, connective tissue; CB, compact bone; IB, immature bone; P, PCL chamber; FC, fibrocartilage; NB, new trabecular bone; NC, new articular cartilage).

The optical images after harvesting the PD‐coated microchamber without spheroids (Chamber), Chamber loaded with PS, and biphasic construct (BS/TS) transplanted tissues showed that the newly generated tissues on PS and BS/TS groups covered the original defect area (dotted line), while that on Chamber group was regenerated weakly (**Figure** **6**a). The X‐ray images of osteochondral tissues from the Defect, Chamber, PS, and BS/TS groups revealed that subchondral bones formed and filled the defect area in the BS/TS implanted group. However, weaker regeneration of bones was found in the Defect and chamber groups (Figure 6b). The 3D images from microcomputed tomography (µCT) investigation showed the tendency more clearly (Figure 6c). The newly formed bone tissue in the Defect group was relatively thin near the cartilage layer, and the subchondral bone layer was regenerated weakly. The bone tissue regenerated in the Chamber and PS groups exhibited features of trabecular bones in the subchondral bone layer, but limited tissue formation was observed near the cartilage layer. Unlike the other groups, the BS/TS group revealed improved formation of trabecular bones in both the subchondral bone and near the cartilage layer. Likewise, the bone volume/total volume (BV/TV) was greatest in the BS/TS group (21.2 ± 2.8%) compared to that from the Defect (9.3 ± 0.5%), Chamber (11.0 ± 1.7%), and PS (12.6 ± 1.4%) groups (Figure 6d). Specifically, the analyses of trabecular bone number (Th.N) (Figure S5a, Supporting Information) and trabecular bone separation (Tb.Sp) (Figure S5b, Supporting Information) indicated that relatively the more segmented, disconnected (islet structured), and underdeveloped trabecular bones were found in the Defect (Th.Sp; 1.5 ± 0.3 mm), Chamber (Th.Sp; 1.4 ± 0.4 mm), and PS (Th.Sp; 1.7 ± 0.4) groups comparing with that of BS/TS group (Th.Sp; 0.3 ± 0.1 mm) while the number of trabecular bones were not significantly different with each other. The H&E staining in the images of the Defect group showed connective tissues in the cartilage area, and the thin and linear compact bones were formed following the tissues, although most of the subchondral bone area was empty (Figure 6e). The images of the Chamber group indicate that the cartilage tissues and lamella structured bone tissues were not formed in the defect area (Figure 6f). In the PS group, trabecular bones and immature bones were formed in the subchondral bone layer, but the only fibrocartilages were found in the cartilage layer (Figure 6g). Unlike the other groups, the images of the BS/TS group showed that the mature articular cartilage and the subchondral bone layer were filled with a large amount of newly formed trabecular bone (Figure 6h).

**Figure 7:**
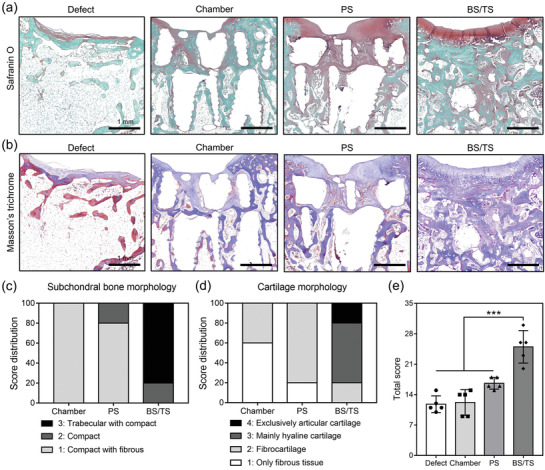
Histological analysis and scoring of rabbit tissues. a) Safranin O and b) Masson's trichrome staining of harvested osteochondral tissues of the Defect, Chamber, PS, and BS/TS groups. Histological scoring for c) subchondral bone or d) cartilage morphology of the regenerated tissues from each group and e) the total scores for each parameter (*n* = 5), and the statistical analysis was performed by one‐way analysis of variance (ANOVA) (*** = *p* < 0.001).

Safranin O staining demonstrated strongly stained articular cartilage only in the BS/TS group (**Figure** **7**a). A large amount of tissue was formed in the cartilage layer of the PS group, although the tissues were immature fibrocartilage that was weakly stained and contained no lacunae structures (Figure 7a). Cartilage tissues (strongly stained in red) were not found in the Defect and C groups (Figure 7a). Masson's trichrome staining demonstrated that most of the tissues regenerated in Chamber, PS, and BS/TS were stained blue, indicating the newly formed osteoid tissues rich in collagens; however, the tissue in the subchondral bone layer of the Defect group mainly was stained red, which shows the lack of active bone regeneration (Figure 7b). Histological scoring for overall defect, subchondral bone, and cartilage of the groups was then performed; more enhanced and host tissue‐like mature regeneration was found in the BS/TS group than in the other groups (Figure S6a–c, Supporting Information). Of the regenerated subchondral bone, 80% was considered trabecular with compact bone, whereas 100% and 80% were compact with fibrous bone in the Chamber and PS groups, respectively (Figure 7c). In the case of cartilage morphology, 20% and 60% of tissues on BS/TS had complete and mainly hyaline cartilage features, respectively, while 40% and 80% of tissues in the Chamber and PS groups were fibrocartilage (Figure 7d). Taken together, the total histological scores were 11.8 ± 1.9, 12.2 ± 2.9, 16.6 ± 1.3, and 25.0 ± 3.7 in the Defect, Chamber, PS, and BS/TS groups, respectively (Figure 7e). The IHC staining of AGG from the sectioned samples of the groups indicated that the signals were clearly shown in the BS/TS group but rarely shown in the other groups (Figure S6d, Supporting Information). In OCN staining, the positive signals were represented strongly in BS/TS transplanted group, weakly shown in PS‐loaded chamber transplanted group, and rarely shown in the other groups (Figure S6d, Supporting Information). Furthermore, the human nucleic antigen (HNA)‐staining of the regenerated tissues demonstrated that 15.1 ± 1.1% and 15.2 ± 0.8% of nuclei were co‐stained with HNA, from the group transplanted with PS and BS/TS, respectively, which were not found in Defect only and no spheroid transplantation groups (Figures S6d and S7, Supporting Information). These results confirmed that the transplanted cells were partially implicated in neo‐cartilage and bone formation.

## 3 Discussion

In this study, we developed composite spheroids of hADSCs containing fibers immobilized with growth factors directing differentiation toward either chondrogenic or osteogenic lineage. Then, we showed that the composite spheroids positioned in a micro‐chamber acted as the cartilage or subchondral bone layer, which was bridged stably without delamination by reunion of sprouting and proliferating hADSCs. Finally, we demonstrated that the transplanted 3D biphasic construct induced regeneration of osteochondral tissue in a rabbit femoral trochlear groove defect model.

The growth factors were immobilized stably on the PD‐coated fibers (Figure S1b, Supporting Information) through chemical reactions of the primary amine and thiol groups in the protein molecules with the *o*‐quinone groups on the PD.^[^ ^29^ ^]^ Similar processes have been reported in previous studies, where BMP‐2 or TGF‐*β* has been immobilized on PD‐coated poly(lactic‐*co*‐glycolic acid) (PLGA)/hydroxyapatite (HA) or a gelatin hydrogel with greater than 80% efficiency.^[^ ^12^, ^30^ ^]^ The immobilized growth factors were active biologically in modulation of proliferation and differentiation of hADSCs, as shown in Figure 1. This was consistent with previous reports that BMP‐2 and TGF‐*β*3 are not only potent regulators of osteogenic and chondrogenic differentiation in MSCs, respectively, but also involved in their proliferation, chemotaxis,^[^ ^31^ ^]^ and pro‐proliferative growth factor secretion.^[^ ^32^ ^]^ In general, the diffusional limitation within a spheroid is caused by the strong cell–cell compaction within large spheroids (>500 µm diameter) and inhibits the penetration of biomolecules. For example, spheroids composed of MSCs or BMSCs cultured in an osteogenic medium containing BMP‐2 showed minimal enhancement of Runx2 expression compared to a spheroid group without inductive factors.^[^ ^33^, ^34^ ^]^ Additionally, the SOX9 expression of MSCs within the spheroid cultured in a chondrogenic medium containing TGF‐*β* was similar to that of the control, indicating that the diffusional limitation should be addressed for effective differentiation of stem cells within spheroids.^[^ ^35^ ^]^ As shown in Figure 1, the gene expression in previous studies was similar to our results (PS/B and PS/T), where the spheroids were stimulated externally during culture in vitro while the growth factors immobilized on the fibers upregulated osteogenic and chondrogenic differentiation of hADSCs more effectively from the inside of BS and TS.

In our previous studies, we demonstrated that fibers decorated with biominerals and platelet‐derived growth factors dramatically enhanced the osteogenic and endothelial differentiation of hADSCs within a spheroid.^[^ ^27^ ^]^ To define the effective doses of BMP‐2 and TGF‐*β*3, the in vitro osteogenic or chondrogenic differentiation was investigated to the spheroids incorporating 0, 50, 100, and 150 ng of each BMP‐2 or TGF‐*β*3 immobilized fragmented fibers (Figure S8, Supporting Information). As a result, the 100 and 150 ng of BMP‐2 significantly enhanced the osteogenic differentiation of hADSCs within a spheroid, but the difference was not found between 100 and 150 ng groups, for example, the 1.4 ± 0.1, 2.1 ± 0.1, and 2.0 ± 0.0 times enhanced OSX expressions were found in the groups incorporating 50, 100, and 150 ng of BMP‐2 than the spheroid without BMP‐2, respectively (Figure S8a, Supporting Information). Similarly, the 100 and 150 ng of TGF‐*β*3 significantly enhanced the chondrogenic differentiation of hADSCs within a spheroid, but the difference was generally not found between 100 and 150 ng groups, for example, the 0.8 ± 0.1, 3.9 ± 0.1, and 2.2 ± 0.2 times enhanced CHAD expressions were found in the groups incorporating 50, 100, and 150 ng of TGF‐*β*3 than the spheroid without TGF‐*β*3, respectively (Figure S8b, Supporting Information). Considering these results, we used 100 ng/spheroid as for the preparation of composite spheroids. Using biomaterials to regulate the phenotype of stem cells directing to chondrocyte^[^ ^9^, ^36^ ^]^ or osteoblast is a promising approach for effective osteochondral tissue regeneration.^[^ ^37^ ^]^ Hwang et al. previously demonstrated that the undifferentiated adipose‐derived stem cells showed 2–3 times downregulated SOX9, AGG, and Col2a gene expressions than the chondrocytes.^[^ ^38^ ^]^ Furthermore, one previous study also demonstrated that the osteoblasts represented three times enhanced RUNX2, OSX, and OCN expressions than the human mesenchymal stem cells.^[^ ^39^ ^]^ Comparing those results with BS and TS, the spheroids showed the similar level of each osteogenic or chondrogenic gene expression than undifferentiated PS group, and the Col1a and Col2a were 6.6 ± 0.2 and 20.4 ± 1.3 times increased, respectively (Figure 1d,f). Thus, it was reasonable to define that the BS and TS were successfully differentiated into osteoblasts and chondrocyte, respectively. Taken together, our results imply that the differentiation of stem cells can be modulated effectively and independently by specific growth factors from inside spheroids cultured in a general growth medium.

Stem cell spheroids have been transplanted for various tissue engineering purposes including vascularization^[^ ^40^ ^]^ and bone and cartilage reconstruction.^[^ ^41^ ^]^ However, the difficulty of localization and severe aggregation of spheroids often limit homogeneous delivery and inhibit natural tissue‐like regeneration.^[^ ^42^ ^]^ Alternatively, spheroids were loaded in a hydrogel for delivery to improve the retention, but the limitation in cell spreading within a gel, difficulty in diffusion of biomolecules, and rapid degradation of a gel in vivo remain problems.^[^ ^43^ ^]^ In contrast, the highly porous structure of 3D‐printed scaffolds could mitigate the diffusion problem by spheroid positioning within the well to prevent aggregation. For example, the spheroids of MSCs and adipose‐derived stromal cells were positioned within a 3D‐printed polyethylene glycol (PEG) modified poly(*ι*‐glutamic acid) scaffold^[^ ^44^ ^]^ and a melt‐electrowritten PCL scaffold,^[^ ^45^ ^]^ and the cells from loaded spheroids showed positive viability. Despite the advantages of a 3D‐printed structure as a carrier of spheroids, the hydrophobicity of PCL is unfavorable for cell adhesion and can allow escape of spheroids from the scaffold during in vivo implantation.^[^ ^46^ ^]^ In this study, we used a mussel‐inspired PD coating method to enhance cell binding affinity, as it has been used commonly on various materials including ceramics, glass, and polymers through *π*–*π* interactions.^[^ ^47^ ^]^ The surface coating not only made the chamber hydrophilic to sink rapidly into the medium but also enhanced cell spreading on the material by providing cell binding moieties through serum‐protein adsorption.^[^ ^48^ ^]^ Thus, the loaded spheroids were retained stably within each chamber (Figure 2b), and the cells from spheroids could proliferate (Figure 2c) and occupy the void spaces of the chamber (Figure 2e).

To enhance the reconstruction of complex osteochondral tissue, pre‐differentiated spheroids composed of MSCs or hADSCs were positioned in an aspiration‐assisted bio‐printed scaffold or chitosan/chitin hydrogel and revealed 12‐fold increase in Runx2 expression and 10 µg of GAG formation, respectively.^[^ ^49^, ^50^ ^]^ Each result was less than that of the cells within BS (Figure 3e) and TS (Figure 3h) organized chambers because the composite spheroids (BS and TS) received instructive signals from the inside of each spheroid. In addition, the effects on differentiation were much greater when each spheroid was positioned within the PD‐coated microchamber (Figure 3). The reason for this could be the increased numbers of cells and cell–cell interactions within a chamber to induce rapid signaling transduction through autocrine and paracrine effects and amplify differentiation.^[^ ^51^ ^]^ Thus, the immobilized BMP‐2 and TGF‐*β*3 on the incorporated fibers within each BS and TS independently induced osteogenic and chondrogenic differentiation of hADSCs in the microchamber and exchanged signals with adherent cells, although each construct was cultured in general medium with no differentiation supplements.

In previous approaches, the delivery of osteogenic (BMP‐2) and chondrogenic (TGF‐*β*3) growth factors has been investigated with the biphasic scaffolds for osteochondral tissue regeneration, however, the reconstruction of the critical sized defect was difficult to be achieved.^[^ ^52^ ^]^ For example, the 2.5 µg mL^−1^ of BMP‐2 or TGF‐*β*3 were covalently bound on a poly(oligo(ethlylene glycol) methacrylate) scaffold to induce osteogenic or chondrogenic differentiation of hMSCs, respectively; however, the in vivo regeneration was not well confirmed.^[^ ^53^ ^]^ Recently, Jiang et al. prepared the bilayered‐silk scaffold loaded with the 200 µg mL^−1^ of BMP‐2 and 20 µg mL^−1^ of TGF‐*β*3, which were transplanted onto the critical sized osteochondral defect of rabbit.^[^ ^54^ ^]^ As a result, the more calcified tissue and mature bone formation were found in the implanted area than the control groups without growth factors; however, empty fissures were observed in the cartilage area and connective tissues were also found in the subchondral bone area.^[^ ^54^ ^]^ The deficient regeneration might be caused by the off‐targeted and inconsistent delivery of growth factors.^[^ ^55^ ^]^ Unlike these approaches, the biphasic construct developed in our approach contained the composite spheroids (BS and TS), which have specifically given the inductive signals to stem cells in a stable manner with reduced amount of BMP‐2 (totally 1.8 µg) or TGF‐*β*3 (totally 0.9 µg) from the inside of a spheroid, showed the more effective differentiation of stem cells (Figure 3), continuously reproduced the growth factors (Figure S4, Supporting Information), and finally succeeded in volumetric regeneration of critical sized osteochondral defect (Figure 7). To prepare a biphasic construct, two scaffolds with different characteristics are integrated through physical adhesion, use of biocompatible glues such as fibrin, or sequential bio‐printing. For example, Lorna et al. physically fused a collagen layer (cartilage phase) with a mineralized collagen layer (bony phase) by a freeze‐drying method,^[^ ^56^ ^]^ and Chen et al. attached a chondrogenic layer composed of a chitosan/gelatin scaffold with an osteogenic layer composed of an HA/chitosan/gelatin scaffold using fibrin glue.^[^ ^57^ ^]^ These approaches succeeded in the partial gluing of two layers, but several apertures were observed between the layers of the construct, which were attributed to in vivo delamination of cartilage and subchondral bone.^[^ ^56^, ^57^ ^]^ The bio‐printing method can resolve this problem since the layers are printed sequentially without separation.^[^ ^17^, ^58^ ^]^ For example, Wang et al. sequentially bio‐printed a silk fibroin biphasic construct using two bio‐inks containing either chondrocytes or BMSCs. However, it was difficult for the printed cells to receive oxygen and instructive signals because of diffusion limitation within the strand, resulting in poor viability and spread.^[^ ^17^ ^]^ Unlike these previous approaches, spontaneously spreading and proliferating hADSCs from each spheroid of TS and BS within the scaffold can act as an adhesive glue (Figure 4) such that the prevalent cell–cell interactions connect the two distinct layers (each BS and TS organized chamber) stably without delamination. Our results imply that fusion by sprouting and proliferating cells can induce the stable joining of cartilage and bony layers without the problems of delamination and compromised cell viability. Although the biphasic constructs developed from previous studies were designed to stimulate chondrogenic or osteogenic differentiation of stem cells (or tissue‐specific cells) loaded in separate layers, uncontrolled differentiation has been reported.^[^ ^59^, ^60^ ^]^ For example, MSCs loaded in a cartilage layer (alginate/hyaluronic hydrogel) were dedifferentiated into osteogenic cells because of intensive osteoinductive signals from an adherent bony layer (BMP‐2 immobilized nanofiber).^[^ ^59^ ^]^ In addition, a previous study of a biphasic scaffold with chondrocyte‐laden hydrogels (cartilage layer) and MSC‐laden PCL scaffolds (bony layer) demonstrated that the majority of stem cells differentiated into chondrocytes because of chondrogenic growth factors secreted from the chondrocytes, which diminished the direction of osteogenesis of stem cells in the bony layer.^[^ ^60^ ^]^ Furthermore, the lamella structured bone was not found after transplanting the same group onto a defect of minipig.^[^ ^60^ ^]^ In contrast, the IHC images of BS/TS demonstrated that the majority of hADSCs from BS loaded area was positive only for Col1a and those from TS only for Col2a because the entrapped BMP‐2 or TGF‐*β*3 within each spheroid induced differentiation independently (Figure 5d,f). Thus, the cells within and spread from BS or TS could retain their osteogenic or chondrogenic characteristics and were not dedifferentiated. The biphasic construct was cultured in general media. The independent spatially controlled differentiation of stem cells was confirmed, as shown in Figure 5d–g, demonstrating that the number of cells positive for Col1a gradually decreased, while that for Col2a gradually increased within the intermediate area (Area 2). These results indicate that sprouting cells retained either chondrogenic or osteogenic lineage from TS or BS, respectively, while forming the interface in the biphasic structure. Taken together, our biphasic scaffold manufactured by hierarchical assembly of spatially organized composite spheroids was not only a new type of therapeutic approach but also solved scientific limitations such as inconsistent delivery of growth factors, delamination of bilayers, insufficient biocompatibility of chemical glue, stem cell dedifferentiation, and limited in vivo volumetric regeneration of critical sized defect.

The bone or cartilage regeneration occurs simultaneously and not independently reproduced following the endochondral ossification steps involving the replacement of hyaline cartilage with bony tissue.^[^ ^61^ ^]^ During this process, the perichondrium surrounding hyaline cartilage becomes infiltrated with blood vessels and osteoblasts, and changes into a periosteum.^[^ ^61^ ^]^ The osteoblasts form a collar of compact bone around the diaphysis, and penetrate the disintegrating cartilage to replace it with spongy bone.^[^ ^62^ ^]^ This forms a primary ossification center, and the ossification continues from this center toward the ends of the bones. When the ossification is complete, the hyaline cartilage is totally replaced by bone except in two edge areas.^[^ ^63^ ^]^ A region of hyaline cartilage remains over the surface of the epiphysis as the articular cartilage.^[^ ^63^ ^]^ The TGF family proteins including the BMP‐2 and TGF‐*β*3 are known to upregulate this endochondral ossification process and cross‐reactivity regardless of their types, however, the BMP‐2 specifically induced final ossification and maturation of osteoblasts to form the bony tissue while the TGF‐*β*3 induced cartilaginous tissue formation and mineralization.^[^ ^64^, ^65^ ^]^ The PCR analysis demonstrated that the TGF‐*β*3 also induced osteogenesis to some extent but not to the extent that of BMP‐2, for example, the Runx2 expression of the cells within TS and BS loaded chambers showed 23.2 ± 6.1 and 56.6 ± 9.1 times greater than that of PS, respectively (Figure S9a, Supporting Information). In contrast, the BMP‐2 also induced chondrogenesis to some extent but not to the extent that of TGF‐*β*3, for example, the AGG expression of the cells from BS and TS organized chambers showed 22.1 ± 2.7 and 52.1 ± 1.5 times greater than that of PS, respectively (Figure S9b, Supporting Information). The light reddish or blue color in TS (alizarin red S staining) or BS (alcian blue staining) loaded area from the biphasic construct could be explained by the weak chondrogenic effect of BMP‐2 and osteogenic effect of TGF‐*β*3 (Figure 5b). In our previous study, we demonstrated that the immobilized growth factors were stably retained on synthetic fibers and entrapped within a composite spheroid, so we are convinced that the regeneration of cartilage and bone was not directed by the leakage of growth factors.^[^ ^66^ ^]^ Taken together, the BMP‐2 and TGF‐*β*3 appeared to show weak cross‐relativity for chondrogenic and osteogenic differentiation of stem cells, respectively; however, the sustained exposure of the hADSCs to BMP‐2 and TGF‐*β*3 within each spheroid directed strong osteogenic and chondrogenic differentiation, respectively.

The in vivo groups were prepared as Defect, Chamber (a commonly used biocompatible scaffold without stem cells), PS (a chamber loads with composite stem cell spheroids without delivery of any inductive signals), and BS/TS (a chamber loaded with stem cell spheroids in which the top and bottom spheroids were prepared with fibers immobilized with TGF‐*β*3 and BMP‐2, respectively). Given that, we would like to test hypotheses that the Chamber group could fill the volume of critical sized defect by guiding the infiltration of tissues and the PS group would enhance regeneration by stem cell transplantation that might solve the problems such as delamination, and the formation of fissures. However, we also anticipated that stem cells without spatially controlled delivery of appropriate inductive signals would not lead to hierarchically assembly of spontaneously differentiating spheroids within chambers, and thus, we hypothesized that the final BS/TS group could induce mature cartilage and subchondral bone formation in a spatially controlled manner. Connective tissues migrate rapidly and cover a defect area in osteochondral tissue to block an infection from external factors, and thin compact bones are formed following a pre‐generated fibrous layer.^[^ ^67^ ^]^ However, this compact bone formed near the cartilage area often disturbs cartilage regeneration because the highly mineralized bony structure extinguishes chondrogenic cell migration and inhibits chondrocyte differentiation and chondral ossification.^[^ ^67^ ^]^ Likewise, regenerated tissues in the Defect group showed CT and an adherent thin compact bone layer (CB) in the cartilage area (Figure 6e), while the internal subchondral layer remained hollow with tissues rarely formed (Figure 6c,e). In contrast, the structures were not found in the chamber‐implanted groups, which indicated that the chamber slowed the rapid infiltration of fibrous tissues in the cartilage area and secured a space for hard tissue regeneration (Figure 6e–h). The use of human stem cells in rabbit skeletal tissue regeneration has been used often and safely without severe side effects; for example, human limbal niche cells, human umbilical cord blood‐derived MSCs, hADSCs, and hBMSCs have been transplanted in rabbit limb, articular cartilage, and bone defect without inflammatory tissue formation.^[^ ^68^, ^69^, ^70^ ^]^ The PLLA fibers have been widely used because of their biocompatibility.^[^ ^71^ ^]^ One study demonstrated that the small amount of PLLA (<10 g) did not reduce pH in physiological environment, nor increase IL‐6 expression of macrophages. ^[^ ^72^ ^]^ In addition, a few hundred mg of PLLA scaffold implanted in the coronary arteries of miniature pig revealed only 1.2 times increased inflammatory and fibrosis score than the control group.^[^ ^73^ ^]^ We previously used similar fibers in various animal and defect models using mouse.^[^ ^28^, ^74^ ^]^ Those in vivo results proved that ≈300 µg of PLLA fibers showed complete degradation within eight weeks without causing any severe inflammatory response in mouse model.^[^ ^66^ ^]^ Similarly, the 270 µg of PLLA fibers were incorporated within the PS or BS/TS groups in this study, and they appeared to be fully degraded after 12 weeks in the defect area, and no sign of inflammatory response stained for multi‐nucleated cells (macrophages) was found (Figure 6g,h). Unlike the small amount of incorporated PLLA fibers, the relatively large volume of PCL chamber might induce the inflammatory response during degradation, which was shown in the H&E staining image of the Chamber group, revealing several inflammatory fibrous tissues (Figure 6f). Interestingly, the inflammatory tissues were not found in the groups of PS and BS/TS containing hADSC spheroids (Figure 6g,h), which may have been due to the previous report that stem cells in mesenchymal affiliation could decrease Th2‐mediated immune response and foreign body reaction.^[^ ^75^, ^76^ ^]^

The most common preclinical osteochondral defect model is the use of the trochlear groove of target animal because it is possible to evaluate both bone and cartilage regeneration from the articular cartilage and subchondral bone structure.^[^ ^77^ ^]^ The critical size of osteochondral defect varies depending on the sizes of animal; for example, 1.4 mm (diameter) × 1.0–2.0 mm (depth) in rat, 3.0–4.0 mm (diameter) × 3.0–5.0 mm (depth) in rabbit, and 4.0 mm (diameter) × 10–12 mm (depth) in dog.^[^ ^77^, ^78^ ^]^ Thus, the cylindrical critical sized defect (4 mm diameter × 3 mm depth) on the femoral trochlear groove of rabbit was created following the previously well‐established surgical procedures.^[^ ^13^, ^79^ ^]^ Stem cell spheroids could be used as building blocks for bone and cartilage by intensively interacting with host tissues while secreting regenerative cytokines and proteins at defect sites.^[^ ^21^, ^41^ ^]^ However, it is difficult to support the maturation of cartilage and bone when spheroids are transplanted without carriers. For example, a previous study transplanted 770 adipose‐derived mesenchymal stromal cell‐spheroids onto an osteochondral defect in vivo without a supportive scaffold and observed a large volume of fissures and formation of bulky fibrocartilage in the defect area. The limited regeneration seems to be caused by failure of stable localization of the spheroids at the defect site.^[^ ^80^ ^]^ To solve this limitation, MSC spheroids were pre‐positioned within carriers such as alginate/hyaluronic hydrogel, decellularized matrix, and 3D‐printed PCL scaffold before transplantation.^[^ ^53^, ^54^ ^]^ However, the regeneration was not improved dramatically because the spheroids could not be differentiated specifically into cartilage or bone without induction of osteogenic or chondrogenic growth factors.^[^ ^83^ ^]^ In our previous study, we demonstrated that both instructive growth factors and carriers for spheroids were necessary for formation of mature bone in a mouse calvarial bone defect.^[^ ^66^ ^]^ Consistent with previous studies, the PS incorporating microchamber transplanted group (with spheroids and without growth factors) showed the more immature bone and fibrocartilages in the defect area than did the Defect and Chamber groups (without spheroids) but showed less mature articular cartilage and lamella structured bones than did the BS/TS group (with composite spheroids incorporating growth factors) group (Figure 7a–d). Furthermore, HNA‐stained nuclei (Figure S6d, Supporting Information) indicated that transplanted human cells within spheroids successfully participated in the process of regeneration.

From histological analysis of the BS/TS group, the void areas where the PCL strands had been located (P; Figure 6h), the small cartilage‐fissures generated during the surgery (Figure 6h), and the HNA‐stained nuclei in the regenerated tissue (Figure S6d, Supporting Information) evidence that the histological cross section was performed precisely at the BS/TS transplanted site. Interestingly, the image of the BS/TS group reveals accelerated degradation of PCL strands relative to the Chamber and PS groups (Figure 7a,b). The rapid degradation of PCL in the BS/TS group might be explained by two mechanisms. First, BMP‐2 and TGF‐*β*3 might have advanced the tissue remodeling process, leading to resorption of damaged tissue and degradation of synthetic materials by immune cells and prevalent hydrolytic enzymes.^[^ ^84^, ^85^ ^]^ Second, the greater number of cells might have induced rapid host tissue ingrowth toward the transplanted chamber that then was replaced rapidly with neo‐tissue by the macrophages participating in the remodeling process.^[^ ^86^, ^87^ ^]^ Previous research found that PCL scaffold seeded with 2 × 10^6^ cells accelerated the degradation of PCL strands relative to that with 1 × 10^6^ cells in a rat calvarial bone defect for eight weeks.^[^ ^87^ ^]^ Taken together, the results of in vivo histological analysis demonstrated that the biphasic construct hierarchically organizing BS and TS simultaneously regenerated large volumes of articular cartilage and trabecular bone.

In summary of the in vivo results, we demonstrated that the more regenerated tissue filled the void defect area in the Chamber group than the Defect only group despite the prevalence of fibrous tissue, and the PS group revealed the more tissue formation than Chamber group while the dual layers were tightly bridged together by the transplanted cell–cell interactions. However, the mature cartilage and bony tissue was rarely founded in both PS and Chamber groups due to the absence of inductive signals. Meanwhile, the BS/TS group showed that the mature articular cartilage formation and the lamella structured trabecular bone tissues localized on each cartilage and subchondral bone area, and the porous spaces from the chamber and fissures were dramatically decreased than the other groups. Furthermore, the transplanted cells from PS and BS/TS groups were stably located with the host cartilage and subchondral bone tissues.

## 4 Conclusions

In this study, we designed a 3D construct carrying composite spheroids of hADSCs that were hybridized with growth factor‐immobilized fibers. The in vitro analysis demonstrated that the uncontrolled differentiation found in stem cells in a biphasic scaffold was resolved with selective TGF‐*β*3 or BMP‐2 signals to hADSCs within composite spheroids. Furthermore, the potential for delamination in a biphasic structure, which can separate the cartilage layer from the subchondral bone region, was addressed since spreading and proliferating cells from each spheroid fused at the intermediate region, forming a strongly attached osteochondral construct. Finally, in vivo transplantation of the biphasic construct demonstrated the combined regeneration of cartilage and bone tissue similar to that of a natural osteochondral tissue‐like structure, which has been difficult to achieve in previous tissue engineering applications. Individually, the PD‐coated microchambers had a role in holding the spheroids on the cartilage or subchondral bone area, and the composite spheroids induced articular cartilage regeneration in the TS and stimulated lamella structured trabecular bone formation in BS. Thick connective tissues in the cartilage area, abnormal cartilaginous tissue, and un‐mineralized tissues in subchondral bone, which are frequent in regenerated osteochondral tissue following transplantation of biphasic constructs, were rare in the BS/TS transplant group. These findings support our 3D biphasic construct with hierarchical assembly of spatially organized composite spheroids and directed induction of chondrogenic and osteogenic differentiation as an advanced therapeutic tool for osteochondral tissue regeneration similar to that of natural cartilage and bone tissues.

## 5 Experimental Section

Experimental details are included in the Supporting Information.

## Conflict of Interest

The authors declare no conflict of interest.

## Supporting information

Supporting Information

## Data Availability

The data that support the findings of this study are available from the corresponding author upon reasonable request.

## References

[1] P. Vashisth , J. R. Bellare , Nanomed.: Nanotechnol., Biol. Med. 2018, 14, 1325.

[2] P. Yeung , W. Zhang , X. N. Wang , C. H. Yan , B. P. Chan , Biomaterials 2018, 162, 1. 29428675 10.1016/j.biomaterials.2018.02.002

[3] M. Maglio , S. Brogini , S. Pagani , G. Giavaresi , M. Tschon , Biomed. Res. Int. 2019, 2019, 4040236. 31687388 10.1155/2019/4040236 PMC6803751

[4] J. Kong , I.‐W. Hwang , K. Lee , Adv. Mater. 2014, 26, 6275. 25043999 10.1002/adma.201402182

[5] Y. Yan , H. Chen , H. Zhang , C. Guo , K. Yang , K. Chen , R. Cheng , N. Qian , N. Sandler , Y. S. Zhang , H. Shen , J. Qi , W. Cui , L. Deng , Biomaterials 2019, 190, 97. 30415019 10.1016/j.biomaterials.2018.10.033

[6] W. Shi , M. Sun , X. Hu , B. Ren , J. Cheng , C. Li , X. Duan , X. Fu , J. Zhang , H. Chen , Y. Ao , Adv. Mater. 2017, 29, 1701089. 10.1002/adma.201701089 28585319

[7] M. T. Frassica , M. A. Grunlan , ACS Biomater. Sci. Eng. 2020, 6, 4324. 33455185 10.1021/acsbiomaterials.0c00753

[8] L.‐P. Yan , J. M. Oliveira , A. L. Oliveira , R. L. Reis , ACS Biomater. Sci. Eng. 2015, 1, 183. 33435045 10.1021/ab500038y

[9] Y. A. Rim , J. H. Ju , Nat. Rev. Rheumatol. 2021, 17, 313. 33753905 10.1038/s41584-021-00604-3

[10] Z. A. Cheng , A. Alba‐Perez , C. Gonzalez‐Garcia , H. Donnelly , V. Llopis‐Hernandez , V. Jayawarna , P. Childs , D. W. Shields , M. Cantini , L. Ruiz‐Cantu , A. Reid , J. F. C. Windmill , E. S. Addison , S. Corr , W. G. Marshall , M. J. Dalby , M. Salmeron‐Sanchez , Adv. Sci. 2019, 6, 1800361. 10.1002/advs.201800361 PMC6343071 30693176

[11] S. Bai , X. Zhang , X. Lv , M. Zhang , X. Huang , Y. Shi , C. Lu , J. Song , H. Yang , Adv. Funct. Mater. 2020, 30, 1908381.

[12] D. Barati , C. Gegg , F. Yang , Ann. Biomed. Eng. 2020, 48, 1971. 32377980 10.1007/s10439-020-02522-z PMC10155292

[13] H. Cho , J. Kim , S. Kim , Y. C. Jung , Y. Wang , B.‐J. Kang , K. Kim , J. Controlled Release 2020, 327, 284. 10.1016/j.jconrel.2020.08.002 32763434

[14] L. Zhou , V. O. GJVM , J. Malda , M. J. Stoddart , Y. Lai , R. G. Richards , K. K.‐w. Ho , L. Qin , Adv. Healthcare Mater. 2020, 9, 2001008. 10.1002/adhm.202001008 33103381

[15] G. W. Fryhofer , H. M. Zlotnick , B. D. Stoeckl , M. J. Farrell , D. R. Steinberg , R. L. Mauck , J. Orthop. Res. 2021, 39, 2323. 33368606 10.1002/jor.24969 PMC8222412

[16] S. Tamburaci , B. Cecen , O. Ustun , B. U. Ergur , H. Havitcioglu , F. Tihminlioglu , ACS Appl. Bio Mater. 2019, 2, 1440. 10.1021/acsabm.8b00700 35026919

[17] D. Kilian , T. Ahlfeld , A. R. Akkineni , A. Bernhardt , M. Gelinsky , A. Lode , Sci. Rep. 2020, 10, 8277. 32427838 10.1038/s41598-020-65050-9 PMC7237416

[18] A. K. Berglund , L. A. Fortier , D. F. Antczak , L. V. Schnabel , Stem Cell Res. Ther. 2017, 8, 288. 29273086 10.1186/s13287-017-0742-8 PMC5741939

[19] N. G. Mehr , X. Li , G. Chen , B. D. Favis , C. D. Hoemann , J. Biomed. Mater. Res., Part A 2015, 103, 2449. 10.1002/jbm.a.35381 25504184

[20] E. Fennema , N. Rivron , J. Rouwkema , C. van Blitterswijk , J. de Boer , Trends Biotechnol. 2013, 31, 108. 23336996 10.1016/j.tibtech.2012.12.003

[21] W. Yang , S. Cai , Y. Chen , W. Liang , Y. Lai , H. Yu , Y. Wang , L. Liu , Adv. Mater. Technol. 2020, 5, 1900847.

[22] N. V. Kosheleva , Y. M. Efremov , B. S. Shavkuta , I. M. Zurina , D. Zhang , Y. Zhang , N. V. Minaev , A. A. Gorkun , S. Wei , A. I. Shpichka , I. N. Saburina , P. S. Timashev , Sci. Rep. 2020, 10, 12614. 32724115 10.1038/s41598-020-69540-8 PMC7387529

[23] P. D. Dalton , T. B. F. Woodfield , V. Mironov , J. Groll , Adv. Sci. 2020, 7, 1902953. 10.1002/advs.201902953 PMC7284200 32537395

[24] S. V. Murphy , P. De Coppi , A. Atala , Nat. Biomed. Eng. 2020, 4, 370. 31695178 10.1038/s41551-019-0471-7

[25] L. M. Allen , J. Matyas , M. Ungrin , D. A. Hart , A. Sen , Stem Cells Int. 2019, 2019, 4607461. 31814836 10.1155/2019/4607461 PMC6878794

[26] S. Kim , E. M. Kim , M. Yamamoto , H. Park , H. Shin , Adv. Healthcare Mater. 2020, 9, 2000608. 10.1002/adhm.202000608 32734719

[27] J. Lee , S. Lee , T. Ahmad , S. K. Madhurakkat Perikamana , J. Lee , E. M. Kim , H. Shin , Biomaterials 2020, 255, 120192. 32559565 10.1016/j.biomaterials.2020.120192

[28] T. Ahmad , H. Byun , J. Lee , S. K. Madhurakat Perikamana , Y. M. Shin , E. M. Kim , H. Shin , Biomaterials 2020, 230, 119652. 31787333 10.1016/j.biomaterials.2019.119652

[29] N. F. Della Vecchia , R. Avolio , M. Alfè , M. E. Errico , A. Napolitano , M. D'Ischia , Adv. Funct. Mater. 2013, 23, 1331.

[30] J. Zhang , J. Li , G. Jia , Y. Jiang , Q. Liu , X. Yang , S. Pan , RSC Adv. 2017, 7, 56732.

[31] M. Wu , G. Chen , Y.‐P. Li , Bone Res. 2016, 4, 16009. 27563484 10.1038/boneres.2016.9 PMC4985055

[32] J. P. Rodríguez , S. Ríos , M. Fernández , J. F. Santibañez , J. Cell. Biochem. 2004, 92, 745. 15211572 10.1002/jcb.20119

[33] N. Miyaguchi , H. Kajiya , M. Yamaguchi , A. Sato , M. Yasunaga , T. Toshimitu , T. Yanagi , A. Matsumoto , H. Kido , J. Ohno , J. Hard Tissue Biol. 2018, 27, 343.

[34] S. K. Min , M. Kim , J.‐B. Park , Exp. Ther. Med. 2020, 20, 79. 32968436 10.3892/etm.2020.9206 PMC7499948

[35] M. C. Goude , T. C. McDevitt , J. S. Temenoff , Cells Tissues Organs 2014, 199, 117. 25413333 10.1159/000365966 PMC4268436

[36] X. Shao , S. Lin , Q. Peng , S. Shi , X. Wei , T. Zhang , Y. Lin , Small 2017, 13, 1602770. 10.1002/smll.201602770 28112870

[37] S. Sirong , C. Yang , T. Taoran , L. Songhang , L. Shiyu , Z. Yuxin , S. Xiaoru , Z. Tao , L. Yunfeng , C. Xiaoxiao , Bone Res. 2020, 8, 6. 32047705 10.1038/s41413-019-0077-4 PMC7010777

[38] J. Lee , C. Y. Lee , J. H. Park , H. H. Seo , S. Shin , B. W. Song , I. K. Kim , S. W. Kim , S. Lee , J. C. Park , S. Lim , K. C. Hwang , Exp. Mol. Med. 2020, 52, 672. 32313200 10.1038/s12276-020-0424-y PMC7210883

[39] T. Eskildsen , H. Taipaleenmaki , J. Stenvang , B. M. Abdallah , N. Ditzel , A. Y. Nossent , M. Bak , S. Kauppinen , M. Kassem , Proc. Natl. Acad. Sci. USA 2011, 108, 6139. 21444814 10.1073/pnas.1016758108 PMC3076836

[40] R. Zhang , W. Luo , Y. Zhang , D. Zhu , A. C. Midgley , H. Song , A. Khalique , H. Zhang , J. Zhuang , D. Kong , X. Huang , Sci. Adv. 2020, 6, eaaz8011. 32494716 10.1126/sciadv.aaz8011 PMC7202876

[41] G. S. Kronemberger , R. A. M. Matsui , G. A. S. C. Miranda , J. M. Granjeiro , L. S. Baptista , World J. Stem Cells 2020, 12, 110. 32184936 10.4252/wjsc.v12.i2.110 PMC7062040

[42] H. Cui , X. Wang , J. Wesslowski , T. Tronser , J. Rosenbauer , A. Schug , G. Davidson , A. A. Popova , P. A. Levkin , Adv. Mater. 2021, 33, 2006434. 10.1002/adma.202006434 PMC11469186 33325613

[43] K. Yanagihara , S. Uchida , S. Ohba , K. Kataoka , K. Itaka , Mol. Ther. 2018, 9, 358. 10.1016/j.omtm.2018.04.006 PMC6054700 30038939

[44] K. Zhang , H. Fang , Y. Qin , L. Zhang , J. Yin , ACS Appl. Mater. Interfaces 2018, 10, 33993. 30207161 10.1021/acsami.8b12268

[45] R. McMaster , C. Hoefner , A. Hrynevich , C. Blum , M. Wiesner , K. Wittmann , T. R. Dargaville , P. Bauer‐Kreisel , J. Groll , P. D. Dalton , T. Blunk , Adv. Healthcare Mater. 2019, 8, 1801326. 10.1002/adhm.201801326 30835969

[46] H. T.‐N. Le , N. B. Vu , P. D.‐N. Nguyen , T. T.‐T. Dao , X. H.‐V. To , P. Van Pham , Front. Biosci.‐Landmark 2021, 26, 266. 10.2741/4894 33049670

[47] S. Hong , Y. Wang , S. Y. Park , H. Lee , Sci. Adv. 2018, 4, 7457. 10.1126/sciadv.aat7457 PMC6128673 30202784

[48] Y. Liu , K. Ai , L. Lu , Chem. Rev. 2014, 114, 5057. 24517847 10.1021/cr400407a

[49] B. Ayan , D. N. Heo , Z. Zhang , M. Dey , A. Povilianskas , C. Drapaca , I. T. Ozbolat , Sci. Adv. 2020, 6, eaaw5111. 32181332 10.1126/sciadv.aaw5111 PMC7060055

[50] V. Zubillaga , A. Alonso‐Varona , S. C. M. Fernandes , A. M. Salaberria , T. Palomares , Int. J. Mol. Sci. 2020, 21, 1004. 10.3390/ijms21031004 PMC7037297 32028724

[51] A. B. Bello , Y. Kim , S. Park , M. S. Muttigi , J. Kim , H. Park , S. Lee , npj Regener. Med. 2021, 6, 50. 10.1038/s41536-021-00160-0 PMC8417285 34480032

[52] S. Abdulghani , P. G. Morouço , J. Mater. Sci.: Mater. Med. 2019, 30, 20. 30689057 10.1007/s10856-019-6218-x

[53] A. Di Luca , M. Klein‐Gunnewiek , J. G. Vancso , C. A. van Blitterswijk , E. M. Benetti , L. Moroni , Biotechnol. J. 2017, 12, 1700072. 10.1002/biot.201700072 28865136

[54] X. Wu , M. Zhou , F. Jiang , S. Yin , S. Lin , G. Yang , Y. Lu , W. Zhang , X. Jiang , Bioact. Mater. 2021, 6, 3976. 33997487 10.1016/j.bioactmat.2021.04.005 PMC8081879

[55] Z. Wang , Z. Wang , W. W. Lu , W. Zhen , D. Yang , S. Peng , NPG Asia Mater. 2017, 9, e435.

[56] B. A. Harley , A. K. Lynn , Z. Wissner‐Gross , W. Bonfield , I. V. Yannas , L. J. Gibson , J. Biomed. Mater. Res., Part A 2010, 92, 1078. 10.1002/jbm.a.32387 19301263

[57] J. Chen , H. Chen , P. Li , H. Diao , S. Zhu , L. Dong , R. Wang , T. Guo , J. Zhao , J. Zhang , Biomaterials 2011, 32, 4793. 21489619 10.1016/j.biomaterials.2011.03.041

[58] A. C. Daly , D. J. Kelly , Biomaterials 2019, 197, 194. 30660995 10.1016/j.biomaterials.2018.12.028

[59] L. Keller , L. Pijnenburg , Y. Idoux‐Gillet , F. Bornert , L. Benameur , M. Tabrizian , P. Auvray , P. Rosset , R. M. Gonzalo‐Daganzo , E. G. Barrena , L. Gentile , N. Benkirane‐Jessel , Nat. Commun. 2019, 10, 2156. 31089136 10.1038/s41467-019-10165-5 PMC6517440

[60] F. Bothe , A.‐K. Deubel , E. Hesse , B. Lotz , J. Groll , C. Werner , W. Richter , S. Hagmann , Int. J. Mol. Sci. 2019, 20, 653. 10.3390/ijms20030653 PMC6387191 30717402

[61] E. J. Mackie , Y. A. Ahmed , L. Tatarczuch , K.‐S. Chen , M. Mirams , Int. J. Biochem. Cell Biol. 2008, 40, 46. 17659995 10.1016/j.biocel.2007.06.009

[62] M. Sarem , M. Heizmann , A. Barbero , I. Martin , V. P. Shastri , Proc. Natl. Acad. Sci. USA 2018, 115, E6135. 29915064 10.1073/pnas.1805159115 PMC6142224

[63] L. Allas , K. Boumédiene , C. Baugé , Bone 2019, 120, 523. 30296494 10.1016/j.bone.2018.10.004

[64] B. Shen , A. Wei , H. Tao , A. D. Diwan , D. D. F. Ma , Tissue Eng., Part A 2009, 15, 1311. 18950289 10.1089/ten.tea.2008.0132

[65] Y. Wang , T. He , J. Liu , H. Liu , L. Zhou , W. Hao , Y. Sun , X. Wang , Mol. Med. Rep. 2016, 14, 5514. 27878265 10.3892/mmr.2016.5961 PMC5355709

[66] J. Lee , J. M. Seok , S. J. Huh , H. Byun , S. Lee , S. A. Park , H. Shin , Biofabrication 2020, 13, 015011. 10.1088/1758-5090/abc39c 33086206

[67] P. Morouço , C. Fernandes , W. Lattanzi , J. Funct. Biomater. 2021, 12, 17. 33673516 10.3390/jfb12010017 PMC7931100

[68] G. Li , Y. Zhang , S. Cai , M. Sun , J. Wang , S. Li , X. Li , S. Tighe , S. Chen , H. Xie , Y. Zhu , Sci. Rep. 2018, 8, 6566. 29700361 10.1038/s41598-018-24862-6 PMC5919904

[69] Y.‐B. Park , C.‐W. Ha , J.‐A. Kim , J.‐H. Rhim , Y.‐G. Park , J. Y. Chung , H.‐J. Lee , PLoS One 2016, 11, e0165446. 27824874 10.1371/journal.pone.0165446 PMC5100912

[70] J. F. Blanco , J. García‐Briñon , L. Benito‐Garzón , D. Pescador , S. Muntión , F. Sánchez‐Guijo , Stem Cells Int. 2018, 2018, 7089484. 30123292 10.1155/2018/7089484 PMC6079361

[71] D. da Silva , M. Kaduri , M. Poley , O. Adir , N. Krinsky , J. Shainsky‐Roitman , A. Schroeder , Chem. Eng. J. 2018, 340, 9. 31384170 10.1016/j.cej.2018.01.010 PMC6682490

[72] C. H. Kum , Y. Cho , Y. K. Joung , J. Choi , K. Park , S. H. Seo , Y. S. Park , D. J. Ahn , D. K. Han , J. Mater. Chem. B 2013, 1, 2764. 32260983 10.1039/c3tb00490b

[73] G. Feng , T. D. Nguyen , X. Yi , Y. Lyu , Z. Lan , J. Xia , T. Wu , X. Jiang , J. Nanomater. 2018, 2018, 1519480.

[74] H. Cho , S. K. Madhurakkat Perikamana , J. Lee , J. Lee , K.‐M. Lee , C. S. Shin , H. Shin , ACS Appl. Mater. Interfaces 2014, 6, 11225. 24942379 10.1021/am501391z

[75] D. Genç , N. Zibandeh , E. Nain , M. Gökalp , A. O. Özen , M. K. Göker , T. Akkoç , Clin. Exp. Allergy 2018, 48, 663. 29498435 10.1111/cea.13126

[76] H. Xia , X. Li , W. Gao , X. Fu , R. H. Fang , L. Zhang , K. Zhang , Nat. Rev. Mater. 2018, 3, 174.

[77] R. Tiwary , Amarpal , H. P. Aithal , P. Kinjavdekar , A. M. Pawde , R. Singh , Cartilage 2014, 5, 43. 26069684 10.1177/1947603513499366 PMC4297094

[78] X. Meng , R. Ziadlou , S. Grad , M. Alini , C. Wen , Y. Lai , L. Qin , Y. Zhao , X. Wang , Biochem. Res. Int. 2020, 2020, 9659412. 32082625 10.1155/2020/9659412 PMC7007938

[79] K. Kim , J. Lam , S. Lu , P. P. Spicer , A. Lueckgen , Y. Tabata , M. E. Wong , J. A. Jansen , A. G. Mikos , F. K. Kasper , J. Controlled Release 2013, 168, 166. 10.1016/j.jconrel.2013.03.013 PMC3661728 23541928

[80] D. Murata , S. Akieda , K. Misumi , K. Nakayama , Tissue Eng. Regener. Med. 2018, 15, 101. 10.1007/s13770-017-0091-9 PMC6171634 30603538

[81] H. Favreau , L. Pijnenburg , J. Seitlinger , F. Fioretti , L. Keller , D. Scipioni , H. Adriaensen , S. Kuchler‐Bopp , M. Ehlinger , D. Mainard , P. Rosset , G. Hua , L. Gentile , N. Benkirane‐Jessel , Nanomed.: Nanotechnol. Biol. Med. 2020, 29, 102253. 10.1016/j.nano.2020.102253 32619705

[82] Y. Teng , X. Li , Y. Chen , H. Cai , W. Cao , X. Chen , Y. Sun , J. Liang , Y. Fan , X. Zhang , J. Mater. Chem. B 2017, 5, 3283. 32264394 10.1039/c7tb00640c

[83] K. Zhang , S. Yan , G. Li , L. Cui , J. Yin , Biomaterials 2015, 71, 24. 26318814 10.1016/j.biomaterials.2015.08.037

[84] J. Jann , S. Gascon , S. Roux , N. Faucheux , Int. J. Mol. Sci. 2020, 21, 7597. 10.3390/ijms21207597 PMC7589189 33066607

[85] X. Xu , L. Zheng , Q. Yuan , G. Zhen , J. L. Crane , X. Zhou , X. Cao , Bone Res. 2018, 6, 2. 29423331 10.1038/s41413-017-0005-4 PMC5802812

[86] K. Yue , G. Trujillo‐de Santiago , M. M. Alvarez , A. Tamayol , N. Annabi , A. Khademhosseini , Biomaterials 2015, 73, 254. 26414409 10.1016/j.biomaterials.2015.08.045 PMC4610009

[87] M. A. Yassin , K. N. Leknes , T. O. Pedersen , Z. Xing , Y. Sun , S. A. Lie , A. Finne‐Wistrand , K. Mustafa , J. Biomed. Mater. Res., Part A 2015, 103, 3649. 10.1002/jbm.a.35505 PMC4744655 26013960

