# Supplementary material for: Directed Regeneration of Osteochondral Tissue by Hierarchical Assembly of Spatially Organized Composite Spheroids
Source: Adv Sci (Weinh). 2021 Nov 21;9(3):2103525. doi: 10.1002/advs.202103525 (PMC8787388; doi:10.1002/advs.202103525)
Supplement: Supplementary file 1 — Supporting Information [file ADVS-9-2103525-s001.pdf]

ADVANCED SCIENCE
Open Access

Supporting Information

for *Adv. Sci.*, DOI: 10.1002/advs.202103525

Directed Regeneration of Osteochondral Tissue by Hierarchical Assembly of Spatially Organized Composite Spheroids

*Jinkyu Lee, Seoyun Lee, Seung Jae Huh, Byung-Jae Kang, and Heungsoo Shin**

Supporting Information

Directed regeneration of osteochondral tissue by hierarchical assembly of spatially organized composite spheroids

*Jinkyu Lee, Seoyun Lee, Seung Jae Huh, Byung-Jae Kang, and Heungsoo Shin**

Experimental Section

Materials: poly(ʟ-lactic acid) PLLA (5.7~8.5 dL/g inherent viscosity, Mn 350~500 kDa) was purchased from Samyang (Seoul, Korea). Dopamine hydrochloride, bis-tris, secondary anti-mouse/rabbit immunoglobulin G (IgG) biotin-conjugated antibody, and alizarin red S were obtained from Sigma (St. Louis, MO, USA). For cell cultures, hADSCs, basal medium, growth supplements, and L-glutamine were purchased from Invitrogen StemPro (Carlsbad, CA, USA). Penicillin/streptomycin (P/S) was obtained from Wisent (St. Bruno, QC, Canada). Phosphate-buffered saline (PBS) and its ion-free version and Dulbecco's phosphate-buffered saline (DPBS) were purchased from Welgene (Gyeongsan, Korea). Tris-HCl and micro bicinchoninic acid (micro-BCA) assay kits were purchased from Alfa Aesar (Haverhill, MA, USA) and Thermo Scientific (Heysham, UK), respectively. H&E solution was purchased from BBC Biochemical (Mount Vernon, MA, USA). The growth factors BMP-2 and TGF-β3 and each protein-related enzyme-linked immunosorbent assay (ELISA) kit were obtained from PeproTech (Rocky Hill, NJ, USA). Fluorescein isothiocyanate (FITC)-streptavidin and mounting medium with diamidino-2-phenylindole (DAPI) were obtained from eBioscience (San Diego, CA, USA) and Vector Laboratories (Burlingame, CA, USA), respectively. Primary antibodies of anti-OCN, OPN, AGG, Col1a, and Col2a were purchased from Abcam (Cambridge, UK).

Preparation of fibers immobilized with growth factors: The fragmented fibers coated with PD were prepared as described in our previous publication.[68] Briefly, aligned PLLA

nanofiber sheets were prepared by electrospinning (5 mL/h, 23G needle, 12~14 keV) using 10 mL of 4% PLLA solution in dichloromethane and trifluoroethanol (8:2, v/v). The electrospun sheets were chopped into small pieces by a mechanical process and chemical aminolysis. Fragmented fibers 60~100 μm in length were collected selectively by sieving and washed sequentially with isopropanol, ethanol, and distilled water (DW). For PD coating, the fibers were immersed in a 2 mg/mL dopamine solution (tris-HCl buffer, pH 8.5) for 20 min. The PD-coated fragmented fibers (PF) were sterilized with 70% ethanol and UV light before use. The PFs (100 μg) were agitated with BMP-2 or TGF-β3 solution (concentration: 1 μg/mL in tris-HCl buffer, 4 ℃, overnight) to synthesize BMP-2 and TGF-β3 immobilized fibers, which were denoted BF and TF, respectively. The morphologies of the fibers were observed by FE-SEM (Hitachi S 4800 FE-SEM, Hitachi; Tokyo, Japan). For FE-SEM imaging, the samples were dehydrated in a 37 ℃ vacuum desiccator for 24 h, attached to an SEM holder by silver paste or carbon tape, and coated with platinum by ion sputter (6 nm/s, 10-15 nm thickness). The amount of growth factors coated on the fibers was quantified indirectly using an ELISA kit. The amount of growth factors in the supernatant of each coating buffer was measured after the immobilization process, and the values were deducted from the initially treated amount of growth factors to calculate the coated amount on the surface of fibers. The surface chemical composition of the fibers was characterized by high-resolution XPS (Theta Probe Base System, Thermo Scientific; Waltham, MA, USA).

Preparation of osteogenic and chondrogenic composite spheroids: The hADSCs were cultured in basal medium with 2% serum, 1% P/S, and 1% L-glutamine under standard culture conditions (5% CO_2 and 37 ℃). Cells were used at passage 4 or 5 throughout all experiments, and the medium was refreshed every 2 days. The hADSCs (40,000) and 10 μg of the engineered fibers were placed in a 0.2 mL tube with 100 μL of growth media to form spontaneously a spheroid within 24 h of centrifugation (1,200 rpm, 5 min). The spheroids

were moved onto a 96-well ultra-unattached cell culture plate (SPL Life Science; Pocheon, Korea) for further *in vitro* culture. The composite hADSC spheroids incorporating fibers of PF, BF, and TF were denoted as PS, BS, and TS, respectively. Furthermore, the PSs cultured with BMP-2 medium (100 ng/mL in growth medium) and TGF-β3 medium (100 ng/mL in growth medium) were denoted PS/B and PS/T, respectively. The PS/B and PS/T were prepared to compare the effect of growth factors delivered from the inside and outside of stem cell spheroids. The shapes of spheroids cultured for 21 days were captured by phase-contrast images through an optical microscope (CKX41, Olympus; Tokyo, Japan), and the sizes of each spheroid were measured from the phase-contrast images. For histological analysis, spheroids cultured for 3 and 21 days were fixed in 4% paraformaldehyde for 20 min, frozen in an optimal cutting temperature (OCT) compound at -80 ℃ overnight, and then cross-sectioned using a cryo-microtome (Cryostat Cryocut, Leica Biosystems GmbH; Wetzlar, Germany). The sectioned specimen was hydrated in sequential steps from 100~70% ethanol and running water. The specimen was stained with hematoxylin for 2 min and with eosin for 8 min. The H&E-stained samples were dehydrated following reverse steps of hydration. FE-SEM images of spheroids were produced after fixation (4% paraformaldehyde), freeze-drying (vacuum, -80 ℃), and the aforementioned SEM sample preparation processes. The DNA assay was performed for the spheroids cultured for 1 and 21 days. The spheroids collected at each time point were lysed in 100 μL of radioimmune precipitation assay (RIPA) lysis buffer (150 mM NaCl, 1% Triton X-100, 1% sodium deoxycholate, 0.1% SDS, 150 mM Tris-HCl, pH 7.2) and reacted with working reagents from a Quant-iT Picogreen dsDNA assay kit (Invitrogen; Carlsbad, CA, USA). The fluorescent intensities with excitation and emission wavelengths of 480 and 520 nm, respectively, from each sample were measured using a spectrophotometer (Varioskan LUX, Thermo Scientific; Waltham, MA, USA).

In vitro differentiation of hADSCs from the spheroids: The spheroids were cultured for 21 days in growth medium to investigate the differentiation of incorporated stem cells. For qPCR (StepOnePlus Real-Time PCR System, Applied Biosystems; Foster City, CA, USA), the mRNA of each spheroid was extracted and purified using a Qiagen RNA extraction kit (Qiagen; Hilden, Germany). The mRNA concentrations were estimated by measuring the absorbance at 260 nm using a nano-spectrometer (Nanodrop 2000, Thermo Scientific; Wilmington, DE, USA). The cDNA was synthesized from 1 μg of each RNA using a Maxime RT PreMix kit (Intron Biotechnology; Seoul, Korea) and a Bio-Rad Thermocycler (Bio-Rad Laboratories; Hercules, CA, USA). The cDNA solution (total volume: 20 μL) was mixed with 10 μL of SYBR® Premix Ex Taq (2×) Tli RNaseH Plus (Takara, Kusatsu, Japan), 0.4 μL of the primers, 0.4 μL of ROX reference dye (50×), and 6.8 μL of diethyl pyrocarbonate (DEPC)-treated RNA-free water (Life Technologies, Carlsbad, CA, USA). The solution was processed in a qPCR instrument for amplification by the following processes; denaturation at 95 °C for 10 min, 40 cycles of annealing at 95 °C for 15 s, extension at 60 °C for 1 min, and melting curve stage at 60.0 °C to 95.0 °C in increments of 0.5 °C per 5 s. The primer sequences were as follows: Runt-related transcription factor 2 (Runx2): forward, 5′-GCA GTT CCC AAG CAT TTC AT-3′, reverse, 5′-CAC TCT GGC TTT GGG AAG AG-3′; OSX: forward, 5′-TAA TGG GCT CCT TTC ACC TG-3′, reverse: 5′-CAC TGG GCA GAC AGT CAG AA-3′, OCN: forward, 5′-GTG CAG AGT CCA GCA AAG GT-3′, reverse, 5′-TCA GCC AAC TCG TCA CAG TC-3′; OPN: forward, 5′-TGA AAC GAG TCA GCT GGA TG-3′, reverse, 5′-TGA AAT TCA TGG CTG TGG AA-3′; Col1a: forward, 5′-CCG GAA ACA GAC AAG CAA-3′, reverse: 5′-AAA GGA GCA GAA AGG GCA-3′; AGG: forward, 5′ - ACA GCT GGG GAC ATT AGT GG-3′, reverse, 5′-GTG GAA TGC AGA GGT GGT TT-3′; SOX9: 5′-GCG GAG GAA GTCGGT GAA GA-3′, reverse, 5′-GTA GAC GGG TTG TTC CCA GT-3′; cartilage oligomeric matrix protein (COMP): forward, 5′-AGG ACA ACT GCG TGA CTG TG-3′, reverse, 5′-GTG TCC TTT TGG TCG TCG TT-3′, chondroadherin

(CHAD): forward, 5′-ACC TGG ACC ACA ACA AGG TC-3′, reverse, 5′-TAG CTG GAC AGC TGG TTC CT-3′; Col2a: forward, 5′-ACA GGG CTC TAA TGA TGT TGA-3′, reverse, 5′-AGG CGT GAT GGC TTA TTT GT-3′, and the housekeeping gene GAPDH: forward, 5′-GTC AGT GGT GGA CCT GAC CT-3′, reverse, 5′-TGC TGT AGC CAA ATT CGT TG-3′. The relative fold changes in gene expression from the PS/B, PS/T, BS, and TS were normalized to that from the PS group. For the calcium assay, the calcium ions deposited in PS, PS/B, and BS were extracted by incubating them in 100 μL of a 0.6 N HCl buffer for 24 h at 37 ℃. Then, 10 μL of the extracted supernatant was mixed with a working reagent from a QuantiChrom calcium assay kit (Bioassay Systems; Hayward, CA, USA), and the optical densities from each sample were measured with a spectrometer at 612 nm. Deposition of calcium minerals in the spheroids was visualized by fixing the cross-sectioned spheroids (following the aforementioned OCT-cryosection method) with 4% paraformaldehyde and then immersing them in an alizarin red S working solution (2% in DW, pH 4.2) for 2 min, followed by washing and mounting. The images were captured by an optical microscope. For the DMMB assay, the PS, PS/T, and TS were lysed in 100 μL of RIPA buffer for 24 h at 4 ℃. The supernatant (20 μL) was mixed with 200 μL of working reagent (16 mg of DMMB, 3.04 g of glycine, 1.6 g of NaCl, 95 ml of 0.1 M acetic acid in 1 L of DW), and the optical densities from each sample were measured with a spectrometer at 525 nm. Deposition of GAGs in the spheroids was visualized by fixing the cross-sectioned spheroids with 4% paraformaldehyde and then immersing them in an alcian blue working solution (1% in 0.1 N HCl with pH 2.5) for 30 min, followed by washing and mounting. The images were captured by an optical microscope.

Preparation of 3D-printed micro-chambers and positioning of spheroids: The method to synthesize the PCL micro-chamber and its mechanical properties are described in our previous manuscript.[69] Briefly, the micro-chambers composed of PCL pellets (Mn 40~50

kDa, cat. 704105, Sigma) were fabricated using a 3D printer constructed at the Korea Institute of Machinery and Materials (KIMM) (Printing conditions: 180~200 mm/min of velocity and 475~500 kPa of pressure). Each strand in the chamber was designed at 200 μm in diameter, and the distance between strands was 300 μm for the first layer and 800 μm for the second to the 10th layers to form square wells using layer-by-layer stacking. The finished chamber had a 5 mm (x-axis) × 5 mm (y-axis) × 1 mm (z-axis) cuboid shape. For PD coating, the micro-chamber was immersed in 2 mg/mL of dopamine buffer (Tris-HCl, pH 8.5, 37 ℃) for 1, 2, 4, and 8 h and rinsed with DW overnight. Optical images of the chambers were collected, and the hydrophilicity of each sample was investigated by dipping the chambers in medium. The surface morphologies of the chambers with or without PD coating were observed by FE-SEM following the aforementioned preparation processes. The surface chemical composition of each chamber was characterized by XPS analysis. For positioning, the spheroids were transferred manually to each well of the chamber one-by-one while being monitored by the microscope. The loading of spheroids using a pipette was manageable. The PS, BS, and TS loaded chambers were denoted C-PS, C-BS, and C-TS, respectively. The samples were cultured for 21 days in growth medium and incubated in a 6-well ultra-unattached cell culture plate (SPL Life Science; Pocheon, Korea). The DNA contents of each sample cultured for 1 or 21 days were lysed using a homogenizer and RIPA buffer to extract the DNA and measured following the aforementioned DNA assay. The morphologies of spheroids positioned within each well of the chambers were captured by an optical microscope after culturing for 1 day, and the cross-sectioned samples cultured for 21 days were stained with H&E. The PCL strands were melted completely during the dehydration steps. The 21-day cultured samples were treated with live/dead assay solution (Molecular Probes; Eugene, OR, USA) to distinguish alive and dead cells, and images were captured by a fluorescent microscope (TE2000, Nikon; Tokyo, Japan).

In vitro differentiation of stem cells from spheroids positioned within the chambers: For alizarin red S and DMMB staining, the samples were fixed for 24 h, dehydrated (with gradient ethanol 70~10% and xylene), and embedded in paraffin blocks. The samples were sectioned horizontally using a microtome (Leica Biosystems GmbH; Wetzlar, Hesse, Germany). The sectioned samples were dipped in xylene to dissolve paraffin, hydrated (reverse steps of dehydration process), and then placed in water. The cross-sectioned PS and BS loaded micro-chambers were stained with alizarin red S, and those of PS and TS specimens were stained with alcian blue working solution. For IHC staining of Col1a and Col2a, the same hydrated and sectioned specimens were fixed with 4% paraformaldehyde and reacted with anti-Col1a for PS and BS loaded chambers and with anti-Col2a for PS and TS loaded chambers (antibody solution; 1:100 in blocking buffer, 0.1% Tween-20, and 5% fetal bovine serum in PBS) for 24 h at 4 ℃, followed by reactions with anti-rabbit IgG biotin-conjugated secondary antibodies (1:100 in PBS) for 1 h at 37 ℃ and FITC-conjugated streptavidin tertiary antibodies (1:100 in PBS) for 1 h at 37 ℃. The stained specimens were mounted with DAPI mounting medium, and images were captured using a fluorescent microscope. The PS and BS organized chambers were homogenized in 300 μL of 0.6 N HCl, and the PS and TS organized chambers were homogenized in 300 μL of RIPA buffer for each calcium and DMMB assay, respectively. After spinning down the debris, the supernatant of each solution was used for the assays following the processes mentioned above. The qPCR analysis was performed for the PS, BS, and TS loaded micro-chambers cultured for 21 days to investigate osteogenic and chondrogenic differentiation of the hADSCs following the aforementioned qPCR process. The samples were lysed in lysis buffer with a homogenizer and incubated for 24 h at 4 ℃ before processing. Furthermore, the culturing media from the chambers carrying PS, BS, and TS were collected during 21 days to analyze the released amount of BMP-2 and TGF-β3 using co-related ELISA kit.

Preparation of biphasic 3D osteochondral construct: The micro-chamber was re-designed in a cylindrical shape with a 4 mm diameter, and the z-axis of the chamber, which was supposed to load the BSs, was changed to 2 mm by stacking of 10 additional layers (total 20 layers). For the subchondral bone layer, 18 BSs were loaded onto the chamber with 2 mm height as the first nine spheroids, and then another nine spheroids were placed on the top of pre-loaded spheroids. For the cartilage layer, nine TS spheroids were loaded onto each well of the chamber with a 1 mm height. Each spheroid loaded within the chamber was cultured for 3 days separately in 6-well ultra-unattached cell culture plates, and then two constructs were stacked so that the BS spheroids contacted the TS spheroids (Figure 5a). The fully integrated 3D construct was denoted as BS/TS and cultured for 21 days to avoid be detachment. The images of the surface and vertically cross-sectioned construct were captured by FE-SEM (the cells in the SEM images were colored green), and the whole construct was observed by a camera while picking up the upper layer (BS loaded chamber) by forceps. The top view of the construct was observed with an optical microscope.

In vitro differentiation of hADSCs from spheroids positioned within the biphasic construct: For histological analysis, the construct was embedded in paraffin following the aforementioned procedure and then sectioned vertically (Figure 6a) or horizontally (Figure 6h) to observe the inside of the construct. The vertically sliced specimen was hydrated and stained with alizarin red S and alcian blue working solutions following the process mentioned above. The specimen was treated with each anti-Co1a and anti-Col2a antibody solution for IHC staining as described above. The numbers of Col1a- or Col2a-positive cells within the red boxes, which indicated cells sprouting from BS and TS in Area 1 and Area 2, respectively, and the intermediate region between chambers in Area 3 were counted. The IHC staining of OPN and SOX9 was performed similarly to that of horizontally sectioned specimens, and the number of antibody-positive nuclei within each well was counted.

In vivo rabbit osteochondral defect model: Twenty male New Zealand white rabbits weighing between 2.5 and 3.0 kg were used for the *in vivo* study. All animals were assigned randomly into four groups: defect without transplantation of chamber (Defect) (n=5), chamber without spheroids (Chamber) (n=5), 27 PS positioned chambers (PS) (n=5), and 9 TS and 18 BS positioned chambers (BS/TS) (n=5). The implants were prepared and pre-cultured *in vitro* over 3 days for stable localization on the chamber and to inhibit the escape of the spheroids. Before surgery, the rabbits were sedated with medetomidine (0.3 mg/kg IM, Domitor, Zoetis; Seoul, Korea) and anesthetized with isoflurane (Ifran, Hana Pharm. Co.; Seoul, Korea) of 3 MAC for induction and 1.5~2 MAC for maintenance in a 1~2 L/min O_2 flow. Under anesthesia, preoperative antibiotics (10 mg/kg, Baytril, Bayer Korea; Seoul, Korea) and analgesics (5 mg/kg, Tramadol HCl, HanAll Biopharma; Seoul, Korea) were injected subcutaneously to minimize discomfort and prevent complications. The surgical site was clipped widely and rinsed with a chlorhexidine-alcohol solution. The skin, subcutaneous tissue, and joint capsule were incised through the lateral parapatellar approach, and the patella was dislocated medially to expose the patellar groove. A cylindrical osteochondral defect of 4 mm in diameter and 3 mm in depth was created on the femoral trochlear groove using a 4 mm drill bit with a drill stopper and a high-speed drill (Colibri II, Depuy Synthes; Oberdorf, Switzerland). During this procedure, the defect site was irrigated with saline and complete hemostasis was achieved by a sterile cotton swab. Then, each defect was implanted with three constructs excluding the Defect only group. Lastly, the joint capsule and subcutaneous tissue were sutured with an absorbable suture (3-0 PDS, Ethicon Inc.; Somerville, NJ, USA), and the skin was closed with a nylon suture (3-0 Blue Nylon, Ailee; Busan, Korea). These surgical procedures were performed on bilateral knees using the same implant. Postoperative management included subcutaneous injection of antibiotics (Baytril; Bayer Korea) and analgesics (Tramadol HCl; HanAll Biopharma) and disinfection of the surgical site with chlorhexidine for 7 days. All procedures for animal experiments were approved by the

Institutional Animal Care and Use Committee (IACUC) of Chaon. Co., Ltd (IACUC No. CE20161). For the control group, the defect was made without transplantation (Defect). At 12 weeks after surgery, the rabbits were anesthetized deeply by 5 MAC of isoflurane and euthanized with intravenous overdose injection of KCl solution (150 mg/kg). The bilateral distal femurs were harvested, trimmed, and fixed in 10% neutral buffered formalin for 3 days. The images of harvested specimens were captured by camera and also analyzed by µCT (Skyscan1176 Bruker microCT) (Billerica, MA, USA) under fixed conditions (x-ray, 60 kV, 417 µA). The 3D images and the BV/TV were examined using a 3D viewer, Ctan, and CTvol software from Bruker µCT. The BV/TV was calculated automatically by Ctan after setting the region of interest (ROI). The TV indicated a fixed value covering all of the initial defect places and was adjusted equally for all samples. Using the same ROI region, the number of trabecular bone (Th.N) and trabecular bone separation (Th.Sp) were also investigated by the Ctan software. All specimens were decalcified in 10% ethylenediamine tetraacetic acid (EDTA) solution (Chelatocal, National Diagnostics; Atlanta, GA, USA) for 4 weeks and trimmed at the center of the defect in a transverse direction. Each sample was dehydrated in ethanol sequentially, embedded in a paraffin block, and cut into 5 µm sections by a microtome. To assess the morphology of repaired tissue, the sections were deparaffinized in xylene; hydrated through graded alcohol to DW; stained with H&E, Masson's trichrome staining (ab150686, Abcam; Cambridge, UK), or safranin O (Samchun Chemical; Pyeongtaek, Korea) with Fast green dye (Fisher Bioreagents; Fair Lawn, NJ, USA) according to a standard protocol; and mounted. For the overall histological scoring of regenerated tissue in the defect, the histological sections were independently scored by 10 evaluators based on an established scoring scheme for osteochondral defects (Table 2).[70,71] The final scores were calculated by averaging the individual scores. For IHC staining of sectioned specimens, anti-AGG and OCN mouse IgG antibodies were used as primary antibodies, and anti-mouse IgG biotin-conjugated antibodies were used as secondary

antibodies. The remaining steps were performed following the IHC procedure for *in vitro* analysis mentioned above, and the images were observed with a fluorescent microscope.

Table 2. Histological scoring system for evaluation of (A) overall tissue filling, (B) subchondral bone repair, and (C) cartilage repair in rabbit osteochondral defects.

Examined categories	Score
(A) Overall defect evaluation (throughout the entire defect depth)	
1. Percentage filling with newly formed tissue	
A. 100%	3
B. > 50%	2
C. < 50%	1
D. 0%	0
2. Percentage degradation of implant	
A. 100%	3
B. > 50%	2
C. < 50%	1
D. 0%	0
(B) Subchondral bone evaluation (within the bottom 2 mm of the defect)	
1. Percentage filling with newly formed tissue	
A. 100%	3
B. > 50%	2
C. < 50%	1
D. 0%	0
2. Subchondral bone morphology	
A. Normal, trabecular bone	4
B. Trabecular bone with some compact bone	3
C. Compact bone	2
D. Compact bone and fibrous tissue	1
E. Only fibrous tissue or no tissue	0
3. Extent of new tissue bonding with adjacent bone	
A. Complete on both edges	3
B. Complete on one edge	2
C. Partial on both edges	1
D. Without continuity on either edge	0
(C) Cartilage evaluation (within the upper 1 mm of the defect)	
1. Morphology of newly formed surface tissues	
A. Exclusively articular cartilage	4
B. Mainly hyaline cartilage	3
C. Fibrocartilage (spherical morphology observed with > 75% of cells)	2
D. Only fibrous tissue (spherical morphology observed with < 75% of cells)	1
E. No tissues	0
2. Thickness of newly formed cartilage	

	A. Similar to the surrounding cartilage	3
	B. Greater than the surrounding cartilage	2
	C. Less than the surrounding cartilage	1
	D. No cartilage	0
3. Joint surface regularity		
	A. Smooth, intact surface	3
	B. Surface fissures (< 25% of new surface thickness)	2
	C. Deep fissures (> 25% of new surface thickness)	1
	D. Complete disruption	0
4. Chondrocyte clustering		
	A. None	3
	B. < 25% chondrocytes	2
	C. 25 ~ 100% chondrocytes	1
	D. No chondrocytes present (no cartilage)	0
5. Chondrocyte and GAG contents of new cartilage		
	A. Normal cellularity with normal Safranin O staining	3
	B. Normal cellularity with moderate Safranin O staining	2
	C. Fewer cells with poor Safranin O staining	1
	D. Few cells with no or little Safranin O staining	0
6. Chondrocyte and GAG contents of adjacent cartilage		
	A. Normal cellularity with normal Safranin O staining	3
	B. Normal cellularity with moderate Safranin O staining	2
	C. Fewer cells with poor Safranin O staining	1
	D. Few cells with no or little Safranin O staining	0

Statistical analysis: All quantitative data were directly calculated and expressed without pre-processing as mean ± SD at least three independent experiments. GraphPad Prism 7 software (La Jolla, CA, USA) was used to perform one-way analysis of variance (ANOVA) followed by Tukey's honestly significant difference test for all investigations except the experiments to compare two of PS, BS, or TS groups, which were analyzed by Student's *t*-test. Significance was denoted as *, **, and *** at a value of $p < 0.05$, $p < 0.01$, and $p < 0.001$, respectively, and all the sample sizes (*n*) were calculated from at least triplicate samples ($n \geq 3$). All the sample sizes and statistical significances were independently denoted on each figure legend.

Supplementary Figures

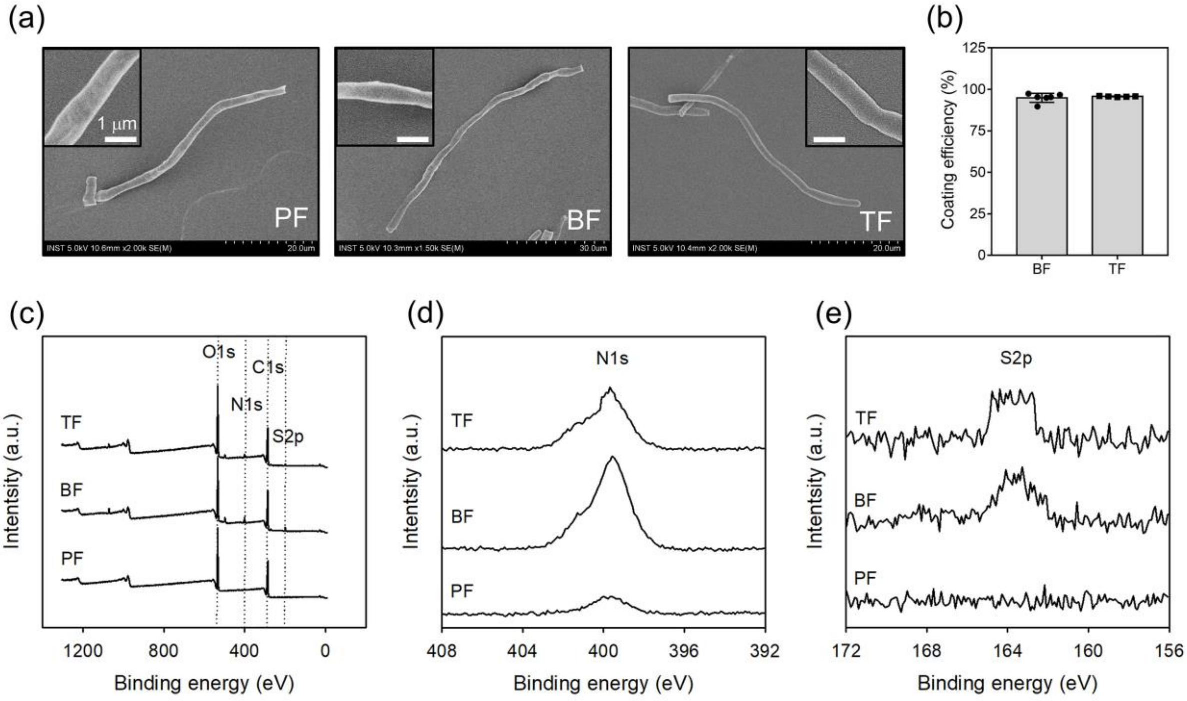

Figure S1. Characterization of the engineered fibers. (a) The morphology and the high-magnified surface of PF, BF, and TF were analyzed by SEM, and (b) the coating efficiencies of BMP-2 and TGFβ-3 on the fibers were measured (*n* = 6). The statistical analysis of coating efficiency was performed by Student's t-test, and they were not shown the significant difference. After the fibers were coated with each growth factor, (c) the overall chemical composition and the high-resolution for (d) N1s or (e) S2p peaks were analyzed by XPS.

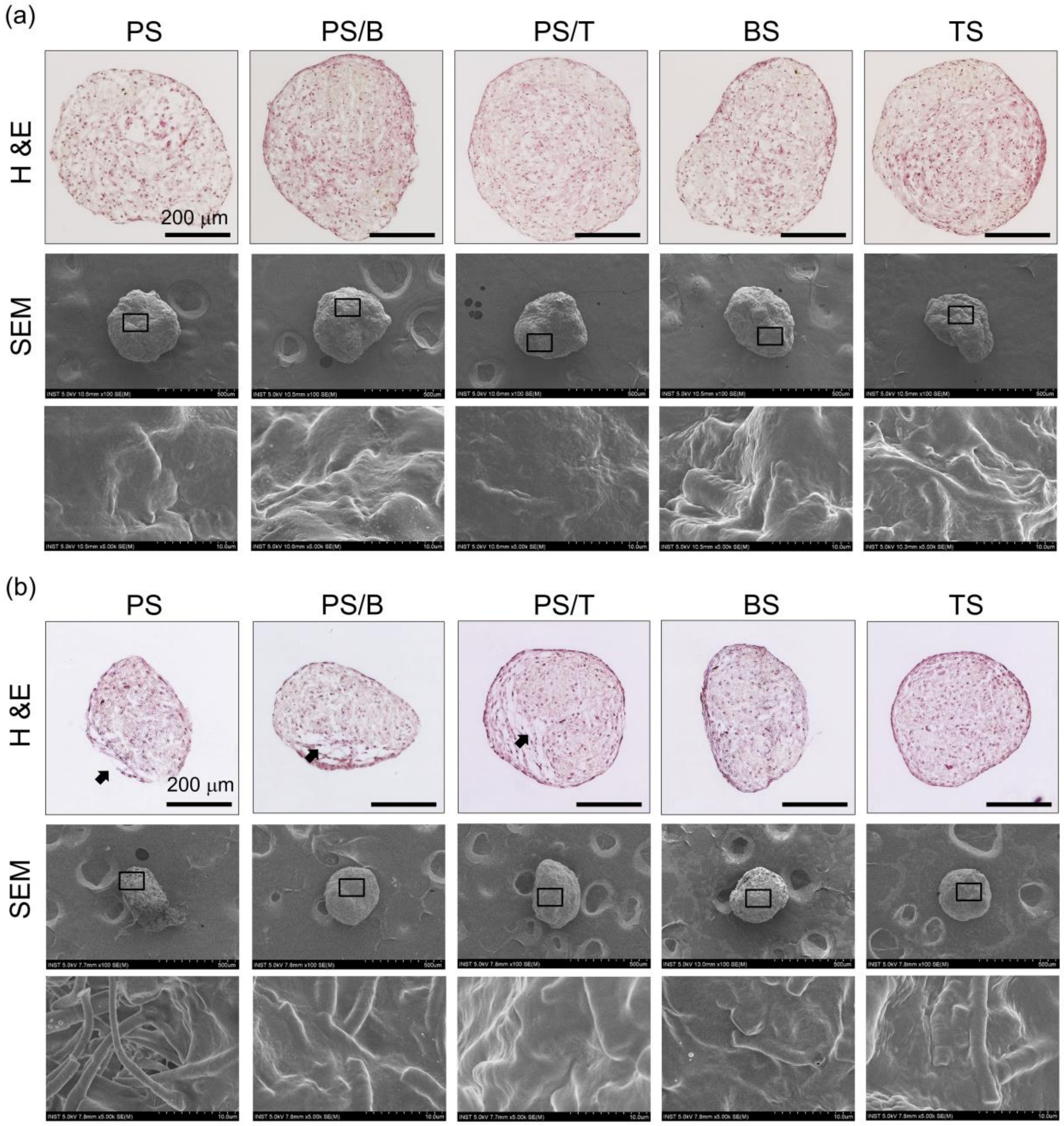

Figure S2. H&E staining and SEM images of spheroids. (a) PS, PS/B, PS/T, BS, and TS cultured for 3 days were stained with H&E after cross-section (scale bar = 200 μm), and the overall morphology of each spheroid and the high-magnified surfaces were observed by SEM. Similarly, (b) the same analysis was performed with each spheroid cultured for 21 days.

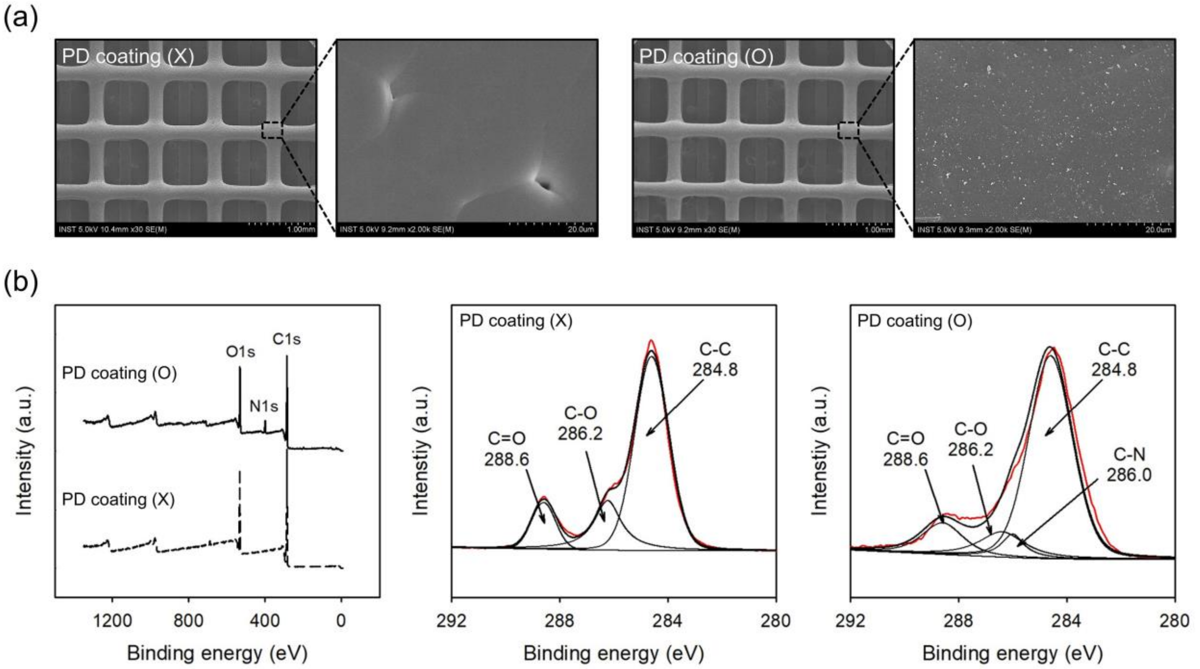

Figure S3. Characterization of a 3D-printed micro-chamber. (a) The lattice shape and the high-magnified surface of micro-chambers before and after PD coating were observed by SEM. (b) The overall chemical composition and the high-resolution of C1s spectra of each chamber were analyzed by XPS.

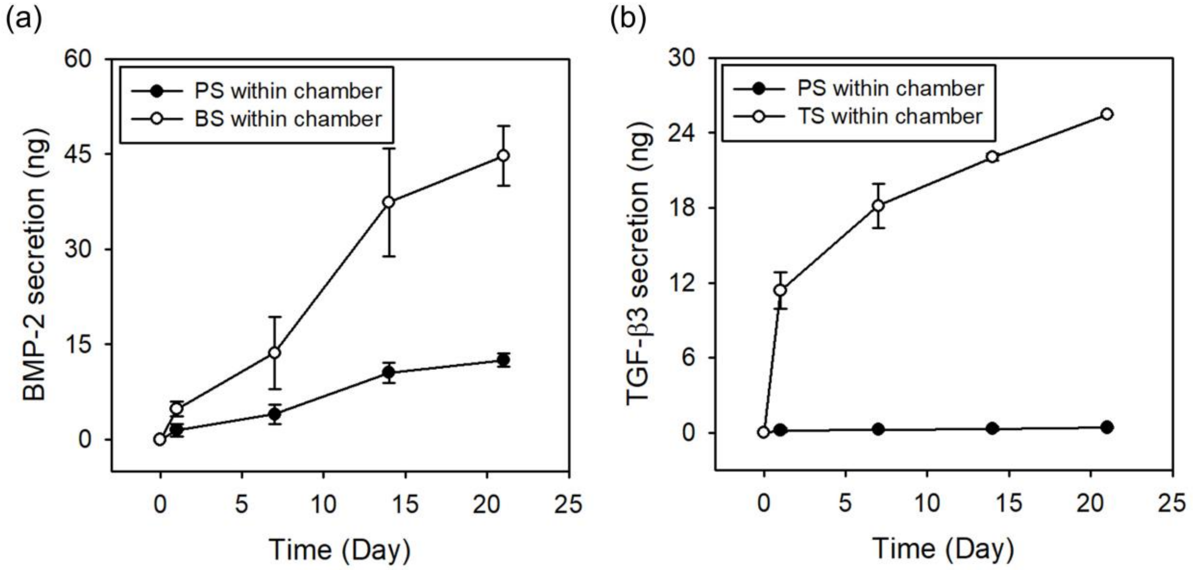

Figure S4. Growth factor secretion from the composite spheroids positioned micro-chambers. The medium culturing the PS, BS, and TS organized chambers were collected during 21 days.

(a) The BMP-2 expressed from the chambers carrying each PS and BS, and (b) the TGF-β3 from the chambers with PS and TS were calculated by each co-related ELISA kit (n = 4 at each time point).

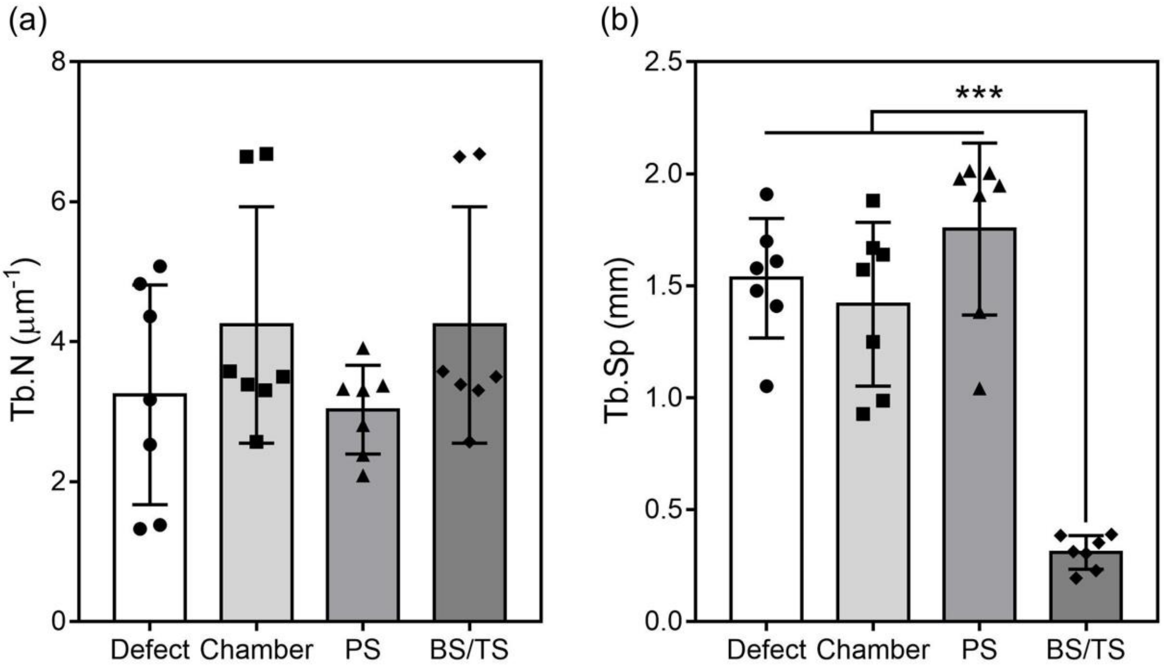

Figure S5. Trabecular bone formation. (a) The number of trabecular bone (Th.N) and (b) trabecular bone separation (Th.Sp) of Defect, Chamber, PS, and BS/TS groups were measured by μCT (n = 7). The statistical analysis was performed by one-way analysis of variance (ANOVA). *** = $p < 0.001$.

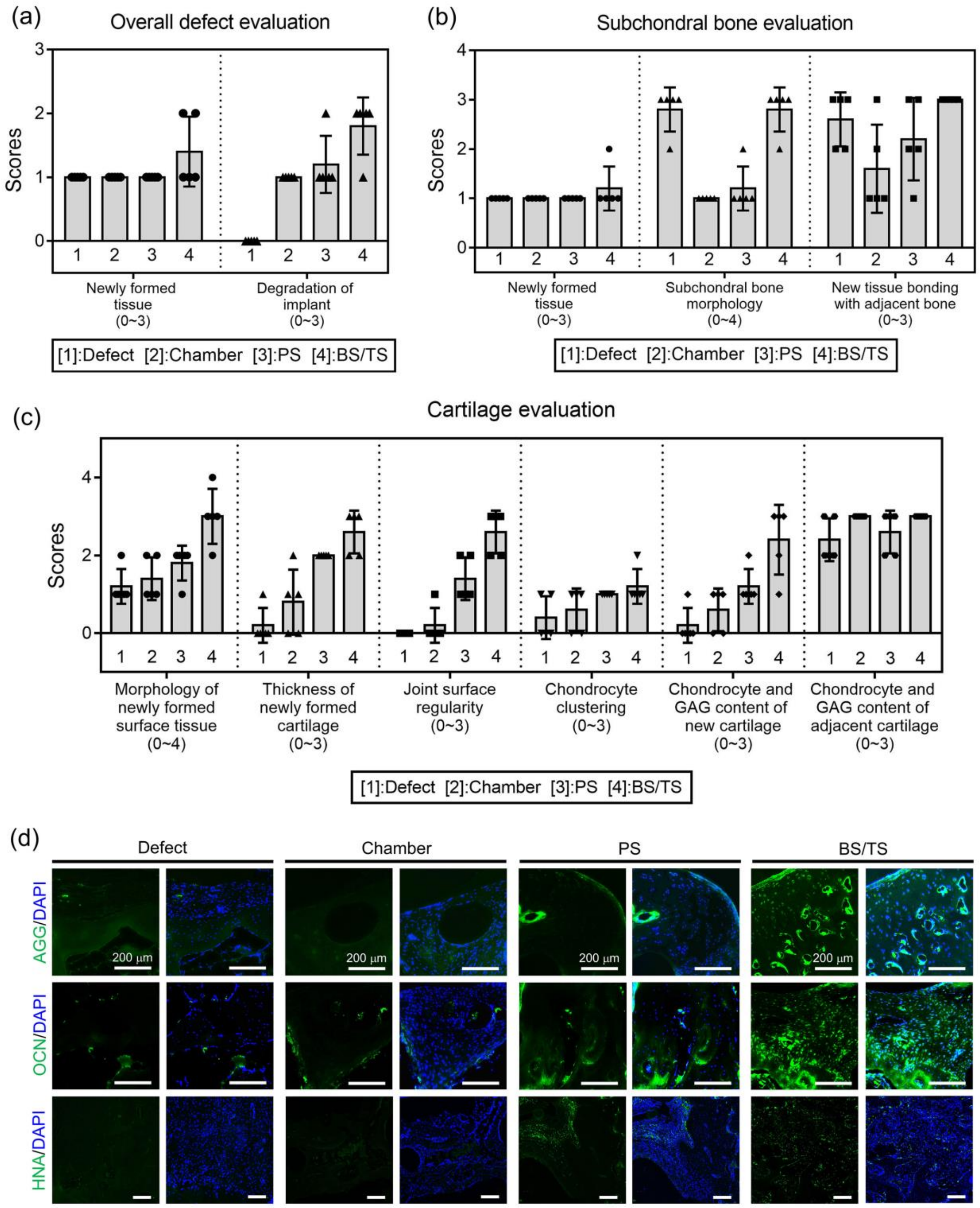

Figure S6. Histological scoring and IHC staining of osteochondral defects. The (a) overall defect, (b) subchondral bone, and (c) cartilage were evaluated following ICRS guidelines after transplanting nothing (Defect), Chamber, PS loaded chamber, and BS and TS loaded biphasic construct (n = 5). (d) The cross-sectioned specimens of the defect area from each group were IHC-stained with AGG, OCN, and HNA.

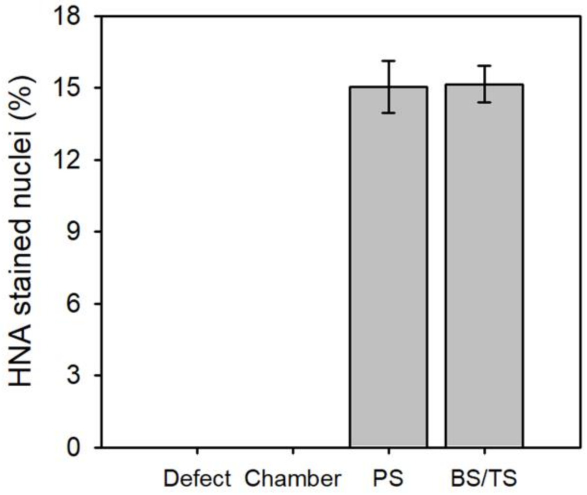

Figure S7. Quantification of HNA stained nuclei (/DAPI stained nuclei) from the cross-sectioned tissue of Defect, Chamber, PS, and BS/TS groups (n = 3).

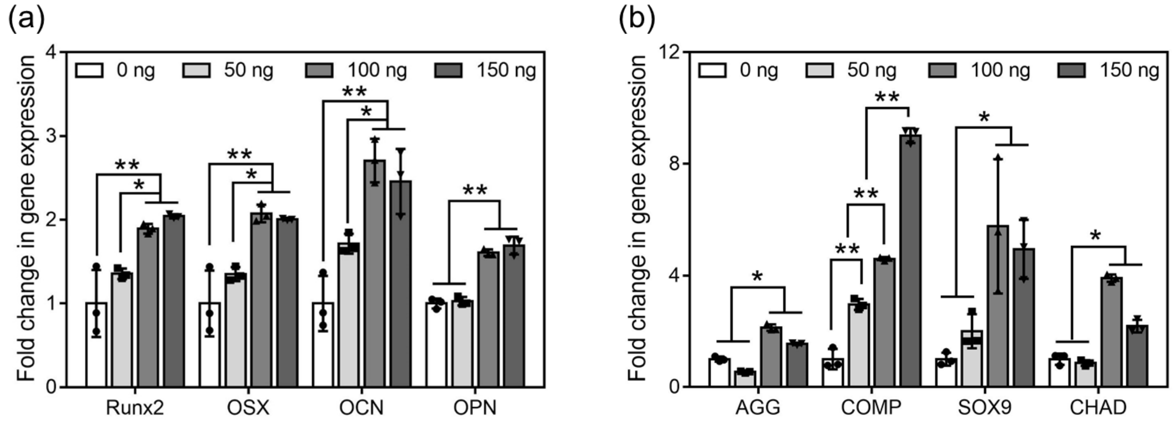

Figure S8. The osteogenic and chondrogenic gene expression of hADSC spheroids incorporating the fibers immobilized with different amounts of growth factors. (a) The PCR analysis of osteogenic gene expression of hADSC spheorids incorporating 0, 50, 100, and 150 ng of BMP-2 immobilized fibers (n = 3), and (b) that of chondrogenic gene expression of hADSC spheroids incorporating 0, 50, 100, and 150 ng of TGF-β3 immobilized fibers (n = 3) after culturing the spheroids within growth medium for 14 days. The statistical analyses were performed by one-way analysis of variance (ANOVA). * = $p < 0.05$ and ** = $p < 0.01$.

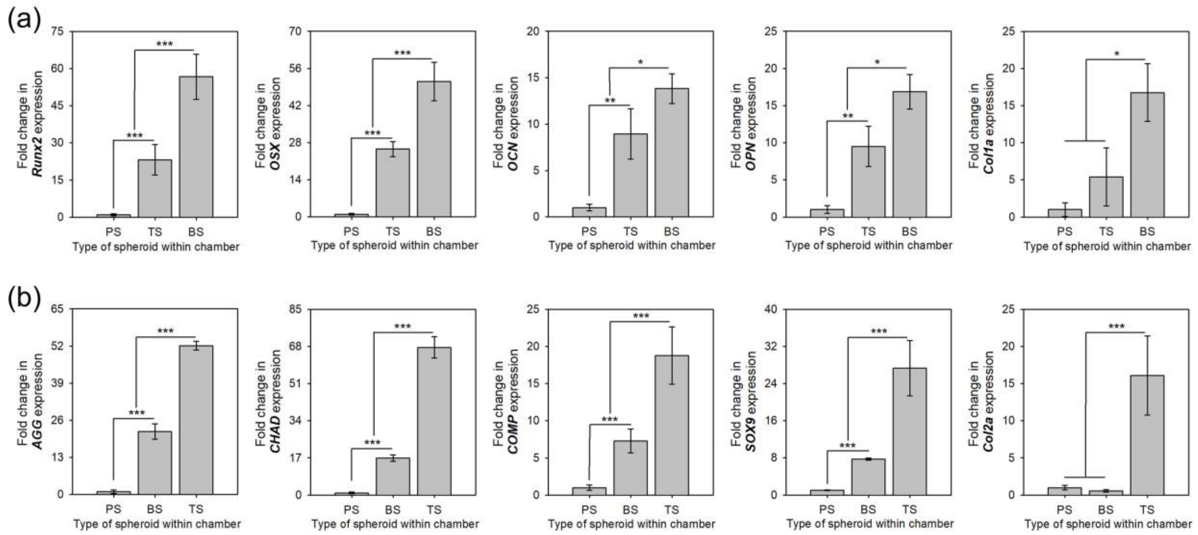

Figure S9. The *in vitro* PCR analysis of osteogenic and chondrogenic gene expression from the composite spheroids laden micro-chambers. (a) Osteogenic gene (Runx2, OSX, OCN, OPN, and Col1a) expressions of PS, TS, and BS loaded micro-chambers, and (b) chondrogenic gene (AGG, CHAD, COMP, SOX9, and Col2a) expressions of PS, BS, and TS organized chambers. The statistical analyses were performed by one-way analysis of variance (ANOVA). * = $p < 0.05$, ** = $p < 0.01$, and *** = $p < 0.001$.
